# Neritinaceramides A–E, New Ceramides from the Marine Bryozoan *Bugula neritina* Inhabiting South China Sea and Their Cytotoxicity

**DOI:** 10.3390/md12041987

**Published:** 2014-04-02

**Authors:** Xiang-Rong Tian, Hai-Feng Tang, Jun-Tao Feng, Yu-Shan Li, Hou-Wen Lin, Xiao-Pei Fan, Xing Zhang

**Affiliations:** 1 Research & Development Center of Biorational Pesticide, College of Plant Protection, Northwest A&F University, Yangling 712100, China; E-Mails: tianxiangrong@163.com (X.-R.T.); fengjt@nwsuaf.edu.cn (J.-T.F.); ting2008ting2008@163.com (X.-P.F.); 2 Institute of Materia Medica, School of Pharmacy, Fourth Military Medical University, Xi’an 710032, China; 3 Department of Pharmacy, Xijing Hospital, Fourth Military Medical University, Xi’an 710032, China; 4 School of Traditional Chinese Medicines, Shenyang Pharmaceutical University, Shenyang 110016, China; E-Mail: liyushan8888@yahoo.com.cn; 5 Department of Pharmacy, Renji Hospital, Affiliated to School of Medicine, Shanghai Jiao-Tong University, Shanghai 200127, China; E-Mail: franklin67@126.com

**Keywords:** marine bryozoan, *Bugula neritina*, ceramide, neritinaceramide, cytotoxicity

## Abstract

Five new ceramides, neritinaceramides A (**1**), B (**2**), C (**3**), D (**4**) and E (**5**), together with six known ceramides (**6**–**11**), two known alkyl glycerylethers (**12** and **13**) and a known nucleoside (**14**), were isolated from marine bryozoan *Bugula neritina*, which inhabits the South China Sea. The structures of the new compounds were elucidated as (2*S*,3*R*,3′*S*,4*E*,8*E*,10*E*)-2-(hexadecanoylamino)-4,8,10-octadecatriene-l,3,3′-triol (**1**), (2*S*,3*R*,2′*R*,4*E*,8*E*,10*E*)-2-(hexadecanoylamino)-4,8,10-octadecatriene-l,3,2′-triol (**2**), (2*S*,3*R*,2′*R*,4*E*,8*E*,10*E*)-2-(octadecanoylamino)-4,8,10-octadecatriene-l,3,2′-triol (**3**), (2*S*,3*R*,3′*S*,4*E*,8*E*)-2-(hexadecanoylamino)-4,8-octadecadiene-l,3,3′-triol (**4**) and (2*S*,3*R*,3′*S*,4*E*)-2-(hexadecanoylamino)-4-octadecene-l,3,3′-triol (**5**) on the basis of extensive spectral analysis and chemical evidences. The characteristic C-3′*S* hydroxyl group in the fatty acid moiety in compounds **1**, **4** and **5**, was a novel structural feature of ceramides. The rare 4*E*,8*E*,10*E*-triene structure in the sphingoid base of compounds **1**–**3**, was found from marine bryozoans for the first time. The new ceramides **1**–**5** were evaluated for their cytotoxicity against HepG2, NCI-H460 and SGC7901 tumor cell lines, and all of them exhibited selective cytotoxicity against HepG2 and SGC7901 cells with a range of IC_50_ values from 47.3 μM to 58.1 μM. These chemical and cytotoxic studies on the new neritinaceramides A–E (**1**–**5**) added to the chemical diversity of *B*. *neritina* and expanded our knowledge of the chemical modifications and biological activity of ceramides.

## 1. Introduction

Ceramides, a family of sphingolipids, are important components of a wide variety of tissues and organs in biological systems [1]. Chemically, ceramides are composed of a sphingoid long chain base (LCB) and an amide-linked long-chain fatty acid base (FAB) [2]. Ceramides and their glycosylated-ceramides (cerebrosides), exhibiting a wide variety of biological effects including cytotoxic, antifungal, immunostimulating and immunosuppressive activities [3], have been isolated from a number of marine invertebrates, including starfishes [4], sea anemones [5,6], sponges [7], corals [8], tunicates [9] and bryozoans [10], as well as terrestrial plants [11]. Marine bryozoans are sedentary, colonial invertebrates that are widely distributed throughout the marine environment, but are less common in freshwater. They have been proved to be an important marine drug sources due to their affluent bioactive secondary metabolites, including macrolide, alkaloids, sterols and heteratom-containing compounds, *etc*., [12]. In particular, a highly oxygenated macrolide, bryostatin 1, isolated previously from marine bryozoan *Bugula neritina*, has been focused on preclinical and clinical studies [13]. In our previous studies focused on marine bryozoans, a new terminal branched ceramide and its analogues [14], cytotoxic sterols [15,16], and bromized alkaloids [17], have been reported from *B. neritina* and *Cryptosula pallasiana*. As part of our ongoing investigations toward the discovery of bioactive secondary metabolites from marine bryozoan *B. neritina*, five new ceramides, neritinaceramides A–E (**1**–**5**), six known ceramides (**6**–**11**), two known alkyl glycerylethers (**12** and **13**), and a known nucleoside (**14**) were isolated. The characteristic C-3′*S* hydroxyl group in the fatty acid moiety in compounds **1**, **4** and **5**, was a novel structure feature of ceramides. The rare 4*E*,8*E*,10*E*-triene structure in the sphingoid base of compounds **1**–**3**, was found from marine bryozoans for the first time. Herein, we report the isolation, structure identification and cytotoxicity evaluation of those new ceramides.

## 2. Results and Discussion

**Figure 1:**
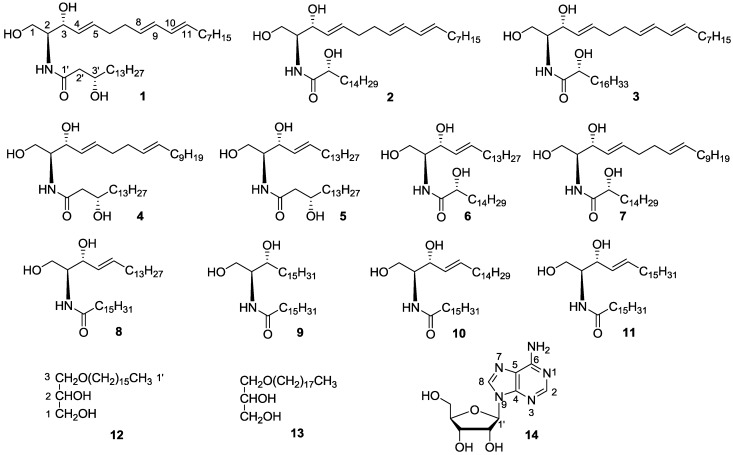
Chemical structures of compounds **1**–**14** from the marine bryozoan *Bugula neritina*.

The marine bryozoan *Bugula neritina* was extracted with 95% EtOH, condensed, and suspended in water, subsequently, and then was further extracted with EtOAc. The EtOAc extract was subjected to column chromatography (CC) over normal silica gel and Sephadex LH-20, respectively, and then was further purified by reverse semi-preparative HPLC to yield compounds **1**–**14** (Figure 1).

**Figure 2:**
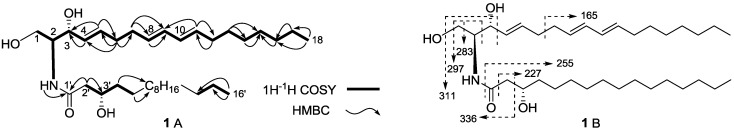
Key ^1^H–^1^H COSY and HMBC correlations (A), together with negative ESI-MS fragments (B) of compound **1**.

Neritinaceramide A (**1**) was isolated as a white amorphous powder. The positive ion mode HR-ESI-MS showed a pseudomolecular ion peak at *m/z* 572.4658 [M + Na]^+^ (calcd. for C_34_H_63_NO_4_Na, 572.4655), which, together with the pseudomolecular ion peak at*m/z* 584 [M + Cl]^−^ in the negative mode ESI-MS, enabled the determination of the molecular formula as C_34_H_63_NO_4_, with the help of NMR spectral data. A close scrutiny of the ^1^H-NMR and ^13^C-NMR spectra of **1** (Table 1) by DEPT and HSQC experiments revealed the presence of an amide group [*δ* 173.0 (C-1′), *δ* 6.65 (1H, *d*, *J* = 8.0 Hz, NH)], a nitrogen-linked methine [*δ* 54.4 (C-2), *δ* 3.92 (1H, *m*, H-2)], a hydroxylated methylene [*δ* 62.0 (C-1), *δ* 3.72 (1H, *br d*, *J* = 8.3 Hz, H-1a), 3.90 (1H, *br d*, *J* = 8.3 Hz, H-1b)] and two hydroxylated methines [*δ* 74.2 (C-3), *δ* 4.31 (1H, *br s*, H-3); *δ* 68.9 (C-3′), *δ* 4.00 (1H, *m*, H-3′)], which together with the long aliphatic carbon signals at 29.2–29.7, demonstrated a trihydroxyl ceramide structure of compound **1**. The observation of ^1^H-^1^H COSY correlations from H_2_-1 to H-2, and in turn, from H-2 to NH and H-3, from H-3′ to H-2a′ (*δ* 2.30), H-2b′ (*δ* 2.43), H-4a′ (*δ* 1.43) and H-4b′ (*δ* 1.51), and the HMBC correlations from NH to C-1′, and from H_2_-2′ to C-1′ and C-3′, indicated the three hydroxyl groups were located at C-1, C-3 an C-3′, respectively. The ^1^H-, ^13^C-NMR and HSQC spectra of **1** suggested the presence of three double bonds (C4=C5, C8=C9 and C10=C11) on the basis of six proton signals at *δ* 5.54 (1H, *dd*, *J* = 15.2, 6.2 Hz, H-4), 5.77 (1H, *dt*, *J* = 15.2, 6.3 Hz, H-5), 5.59 (1H, *m*, H-8), 5.99 (1H, *dd*, *J* = 15.3, 5.0 Hz, H-9), 6.00 (1H, *dd*, *J* = 15.3, 5.0 Hz, H-10) and 5.52 (1H, *m*, H-11), as well as the corresponding carbons at *δ* 129.3 (C-4), 133.2 (C-5), 133.3 (C-8), 130.0 (C-9), 131.2 (C-10) and 130.7 (C-11). The positions of the three double bonds were confirmed to be C-4, C-8 and C-10 on the basis of ^1^H-^1^H COSY correlations of H-3/H-4, H-4/H-5, H-5/H_2_-6, H_2_-6/H_2_-7, H_2_-7/H-8, H-8/H-9, H-9/H-10 and H-10/H-11, and HMBC correlations of H-4/C-3, H-5/C-3, H-5/C-6, H_2_-6/C-4, H_2_-6/C-5, H_2_-6/C-7, H_2_-7/C-8, H_2_-7/C-9, H-8/C-10, H-11/C-9 and H-11/C-12 (Figure 2A). The lengths of the sphingoid long chain base (LCB) and the amide-linked long-chain fatty acid base (FAB) were determined to be composed of 18 and 16 carbons, respectively, based on the negative ESI-MS fragment ions at *m/z* 165, 227, 255, 283, 297, 311 and 336 (Figure 2B). Based on the above evidences, the planar structure and the key connectivities of ceramide **1** were established.

**Table 1:** ^1^H and ^13^C-NMR data of compounds **1** and **2** (500MHz for ^1^H-NMR, 125MHz for ^13^C-NMR) ^a^.

No.	1		2		
	*δ*_C_, *mult.* ^b^	*δ*_H_ (int., *mult.*, *J* in Hz) ^b^	*δ*_C_, *mult.* ^b^	*δ*_C_, *mult.* ^c^	*δ*_H_ (int., *mult.*, *J* in Hz) ^c^
1	62.0 (*t*)	3.72 (1H, *br d*, 8.3), 3.90 (1H, *br d*, 8.3)	62.0 (*t*)	61.9 (*t*)	4.22 (1H, *dd*, 10.8, 4.2), 4.46 (1H, *dd*, 10.8, 4.5)
2	54.4 (*d*)	3.92 (1H, *m*)	54.4 (*d*)	56.1 (*d*)	4.67 (1H, *m*)
3	74.2 (*d*)	4.31 (1H, *br s*)	74.2 (*d*)	73.0 (*d*)	4.83 (1H, *br t*, 6.1)
4	129.3 (*d*)	5.54 (1H, *dd*, 15.2, 6.2)	129.1 (*d*)	131.4 (*d*)	6.13 (1H, *m*)
5	133.2 (*d*)	5.77 (1H, *dt*, 15.2, 6.3)	133.3 (*d*)	132.6 (*d*)	6.05 (1H, *m*)
6	31.9 (*t*)	2.16 (2H, *m*)	31.9 (*t*)	32.7 (*t*)	2.18 (1H, *m*)
7	32.1 (*t*)	2.04 (2H, *dt*, 14.1, 6.9)	32.1 (*t*)	32.9 (*t*)	2.04 (1H, *m*)
8	133.3 (*d*)	5.59 (1H, *m*)	133.5 (*d*)	132.8 (*d*)	5.64 (1H, *m*)
9	130.0 (*d*)	5.99 (1H, *dd*, 15.3, 5.0)	130.0 (*d*)	131.0 (*d*)	6.11 (1H, *m*)
10	131.2 (*d*)	6.00 (1H, *dd*, 15.3, 5.0)	131.2 (*d*)	131.6 (*d*)	5.65 (1H, *m*)
11	130.7 (*d*)	5.52 (1H, *m*)	130.7 (*d*)	131.5 (*d*)	5.99 (1H, *m*)
12	32.1 (*t*)	2.16 (2H, *m*)	32.1 (*t*)	32.7 (*t*)	2.18 (2H, *br s*)
13	32.6 (*t*)	2.04 (2H, *dt*, 14.1, 6.9)	32.6 (*t*)	32.1 (*t*)	1.23 (2H, *br s*) ^d^
14	29.4 (*t*)	1.36 (2H, *m*) ^d^	29.4 (*t*)	29.4 (*t*)	1.33 (2H, *m*) ^d^
15	29.2 (*t*)	1.36 (2H, *m*) ^d^	29.2 (*t*)	29.3 (*t*)	1.33 (2H, *m*) ^d^
16	31.9 (*t*)	1.26 (2H, *br s*) ^d^	31.9 (*t*)	32.1 (*t*)	1.23 (2H, *br s*) ^d^
17	22.7 (*t*)	1.26 (2H, *br s*) ^d^	22.7 (*t*)	22.9 (*t*)	1.23 (2H, *br s*) ^d^
18	14.1 (*q*)	0.88 (3H, *t*, 7.1)	14.1 (*q*)	14.2 (*q*)	0.84 (3H, *t*, 7.1)
1′	173.0 (*s*)	-	175.0 (*s*)	175.4 (*s*)	-
2′	43.1 (*t*)	a 2.30 (1H, *m*), b 2.43 (1H, *d*, 2.2)	72.4 (*d*)	72.5 (*d*)	4.59 (1H, *m*)
3′	68.9 (*d*)	4.00 (1H, *m*)	34.8 (*t*)	35.8 (*t*)	2.22 (1H, *m*), 2.03 (1H, *m*)
4′	37.1 (*t*)	a 1.43 (1H, *m*), b 1.51 (1H, *m*)	31.8 (*t*)	32.0 (*t*)	1.23 (2H, *br s*) ^d^
5′	25.6 (*t*)	1.37 (2H, *m*)	25.1 (*t*)	25.9 (*t*)	1.64 (2H, *m*)
6′~13′	29.2–29.7 (*t*)	1.26 (16H, *br s*) ^d^	29.2–29.7 (*t*)	29.3–30.0 (*t*)	1.23 (16H, *br s*) ^d^
14′	31.9 (*t*)	1.26 (2H, *br s*) ^d^	31.9 (*t*)	32.1 (*t*)	1.23 (2H, *br s*) ^d^
15′	22.7 (*t*)	1.26 (2H, *br s*) ^d^	22.7 (*t*)	22.9 (*t*)	1.23 (2H, *br s*) ^d^
16′	14.1 (*q*)	0.88 (3H, *t*, 7.1)	14.1 (*q*)	14.2 (*q*)	0.84 (3H, *t*, 7.1)
NH	-	6.65 (1H, *d*, 8.0)	-	-	8.34 (1H, *d*, 8.7)

^a^ Assignments aided by the DEPT, COSY, HSQC, HMBC, and NOESY experiments; ^b^ data were obtained in CDCl_3_; ^c^ data were obtained in C_5_D_5_N; ^d^ overlapped with other signals.

**Figure 3:**
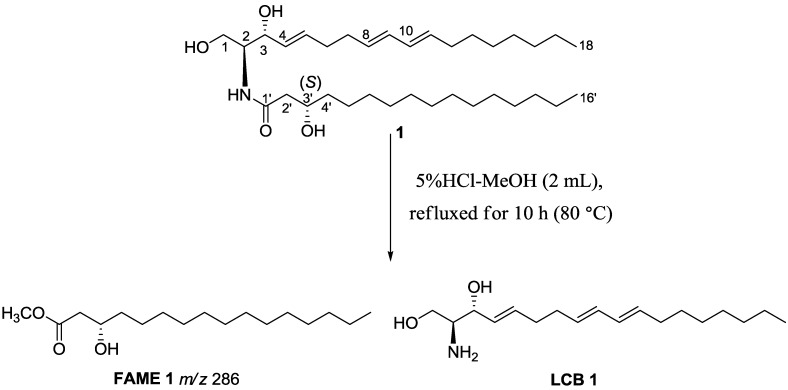
Methanolysis of compound **1**.

The geomertry of the C-4/C-5, C-8/C-9 and C-10/C-11 alkenyl bonds was confirmed to be *trans* based on the vicinal coupling constants of *J*_4,5_ (15.2 Hz), *J*_8,9_ (15.3 Hz) and *J*_9,10_ (15.3 Hz). Moreover, the *trans* configuration (*E*) for those alkenyl bonds was further confirmed by the chemical shifts of the allylic carbons C-6 (*δ* 31.9), C-7 (*δ* 32.1) and C-12 (*δ* 32.1), since allylic carbon signals of *trans*- and *cis*-isomers were observed at *δ* 32–33 and 27–28, respectively [18]. The relative stereochemistry at C-2 and C-3 in LCB of **1** were found to be same as that of d-sphingosine (d-*erythro*), as evidenced by ^13^C-NMR chemical shifts of C-2 (*δ* 54.4) and C-3 (*δ* 74.2), which are consistent with those reported for natural product *N*-palmitoyl-d-*erythro*-(2*S*,3*R*)-octadecasphinga-4(*E*)-ene (*δ* 54.5 and 74.7) [19]. Furthermore, the specific rotation ([α]_D_ = −2.8°) of LCB **1** obtained by methanolysis of ceramide **1**, was in good accordance with d-*erythro*-sphingosine, (2*S*,3*R*)-(*E*)-2-aminooctadec-4-ene-1,3-diol ([α]_D_ = −3.0°) [20], while different from l-*erythro*-sphingosine, (2*R*,3*S*)-(+)-*erythro*-*N*-lauroyldocosasphinga-4,8-dienine ([α]_D_ = +2.94°) [21]. This further confirmed the 2*S*,3*R* configuration for ceramide **1**. Similarly, the 3′*S*-configuration was proved by the methanolysis of **1** and comparison of the specific rotation of the resulting methyl ester of the fatty acid with known compounds. In details, methanolysis of **1** by hydrochloric acid/methanol gave fatty acid methyl ester (FAME **1**) as the major product (Figure 3). The specific rotation of FAME **1** ([α]_D_ +12.5°) was in good agreement with the synthetic (*S*)-methyl 3-hydroxyhexadecanoate ([α]_D_ = +13.8°), while the specific rotation [α]_D_ for (*R*)-methyl 3-hydroxyhexadecanoate was −16.6° [22]. Thus, the structure of neritinaceramide A was unambiguously assigned as (2*S*,3*R*,3′*S*,4*E*,8*E*,10*E*)-2-(hexadecanoylamino)-4,8,10-octadecatriene-l,3,3′-triol.

**Figure 4:**
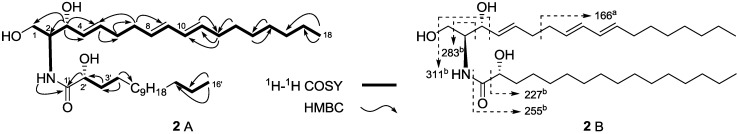
Key ^1^H-^1^H COSY and HMBC correlations (**A**), together with ESI-MS fragments (**B**) of compound **2** (^a^ positive fragment ions; ^b^ negative fragment ions).

Neritinaceramide B (**2**) was isolated as an isomer of **1**, due to the same molecular formula of C_34_H_63_NO_4_ from ESI-MS at *m/z* 572 [M + Na]^+^ (positive mode) and 584 [M + Cl]^−^ (negative mode), and the HR-ESI-MS pseudomolecular ion peak at *m/z* 572.4652 [M + Na]^+^ (calcd. for C_34_H_63_NO_4_Na, 572.4655). By comparison of the ^13^C-NMR data of **2** with those of **1**, it could be confirmed that **2** and **1** shared the same LCB structural skeleton, while the hydroxyl group at C-3′ in ceramide **1** was replaced to be at C-2′ in **2**, as **2** showed different chemical shifts at C-1′ (*δ* 175.4), C-2′ (*δ* 72.5) and C-3′ (*δ* 35.8) in its FAB. The methene protons H_2_-3′ (*δ* 2.03 and 2.22) showed coupling relationships with H_2_-4′ (*δ* 1*.*23) and H-2′ (*δ* 4.59) in the ^1^H-^1^H COSY spectrum, which together with HMBC correlations from NH (*δ* 8.34, *d*, *J* = 8.7 Hz) to C-1′, from H-2′ to C-1′ and from H_2_-3′ to C-2′, confirmed that the hydroxyl group was attached at C-2′ (Figure 4A). Fragment peaks at *m/z* 166, 227, 255, 283 and 311 indicated that the number of carbons in the sphingoid long chain base was 18, and in the fatty acid base was 16 (Figure 4B). The stereochemistry at C-2, C-3 and C-2′ were proposed as 2*S*, 3*R* and 2′*R* on the basis of the chemical shifts of C-2, C-3, and C-2′ in CDCl_3_ (*δ* 54.4, 74.2 and 72.4, respectively) and C_5_D_5_N (*δ* 56.1, 73.0 and 72.5, respectively), which were close to analogue (2*S*,3*R*,4*E*,2′*R*)-2-(2-hydroxyhexadecanoylamino)-16-methyl-4-octadecene-1,3-diol [*δ* 54.6 (C-2), 74.3 (C-3) and 72.5 (C-2′) in CDCl_3_, and *δ* 56.1 (C-2), 73.1(C-3) and 72.5 (C-2′) in C_5_D_5_N.] [23]. Moreover, the configurations were confirmed by the specific rotation of ceramide **2** ([α]_D_ = +9.8°) which was consistent with that of the synthetic ceramide with the 2*S*,3*R*,2′*R*-configuration ([α]_D_ = +7.4°) [24], and this method has been applied to determine the stereochemistry for ceramide with the core structure of 2,3,2′ chiral centre [25,26]. Besides, methanolysis of **2** with the same method of **1** gave fatty acid methyl ester (FAME **2**), its specific rotation ([α]_D_ = –6.7°) was similar to (*R*)-methyl 2-hydroxyhexadecanoate ([α]_D_ = –5.7°) [26], while was different with (*S*)-methyl 2-hydroxyhexadecanoate ([α]_D_ = +1.15°) [27], which also confirmed the 2′*R*-configuration for ceramide **2**. Accordingly, the structure of neritinaceramide B (**2**) was deduced as (2*S*,3*R*,2′*R*,4*E*,8*E*,10*E*)-2-(hexadecanoylamino)-4,8,10-octadecatriene-l,3,2′-triol.

**Figure 5:**
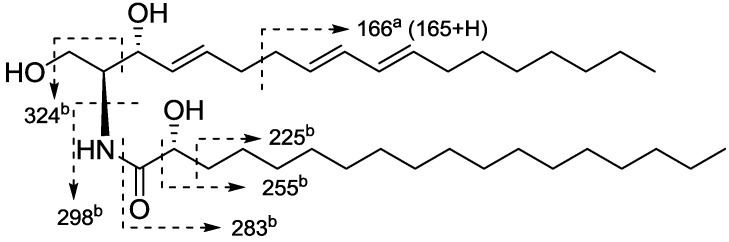
Key ESI-MS fragments of compound **3** (^a^ positive fragment ions; ^b^ negative fragment ions).

Neritinaceramide C (**3**) was found to have a molecular formula C_36_H_67_NO_4_ according to the positive ESI-MS pseudomolecular ion peak at *m/z* 600 [M + Na]^+^, and negative ESI-MS pseudomolecular ion peak at *m/z* 576 [M − H]^−^, with the help of NMR spectral data (Table 2). The NMR spectra of **3** showed a close resemblance to those of ceramide **2**, except for the carbon number in the FAB. The negative ESI-MS fragment ions at *m/z* 324, 298, 283, 255 and 225 indicated that the numbers of carbons for both of the FAB and LCB were 18 (Figure 5). The carbon number of the FAB was further validated through EI-MS molecular ion at *m/z* 314 (M^+^) for the methyl ester obtained after methanolysis of **3** with 5% mehtanolic HCl. Therefore, neritinaceramide C (**3**) was determined to be (2*S*,3*R*,2′*R*,4*E*,8*E*,10*E*)-2-(octadecanoylamino)-4,8,10-octadecatriene-l,3,2′-triol.

**Table 2:** ^1^H and ^13^C-NMR data of compounds **3**–**5** (CDCl_3_, 500MHz for ^1^H-NMR, 125MHz for ^13^C-NMR) ^a^.

No.	3		4		5	
	*δ*_C_, *mult.*	*δ*_H_ (int., *mult.*, *J* in Hz)	*δ*_C_, *mult.*	*δ*_H_ (int., *mult.*, *J* in Hz)	*δ*_C_, *mult.*	*δ*_H_ (int., *mult.*, *J* in Hz)
1	62.2 (*t*)	3.75 (1H, *m*), 3.89 (1H, *br d*, 10.0)	61.8 (*t*)	3.71 (1H, *d*, 11.0), 3.86 (1H, *d*, 11.2)	61.9 (t)	3.74 (1H, *m*), 3.87 (1H, *m*)
2	54.4 (*d*)	3.91 (1H, *br d*, 10.0)	54.5 (*d*)	3.92 (1H, *m*)	56.1 (*d*)	3.93 (1H, *m*)
3	74.2 (*d*)	4.28 (1H, *m*)	74.1 (*d*)	4.28 (1H, *br s*)	74.1 (*d*)	4.28 (1H, *m*)
4	129.2 (*d*)	5.54 (1H, *m*)	128.9 (*d*)	5.51 (1H, *dd*, 15.5, 6.0)	128.6 (*d*)	5.53 (1H, *br d*, 14.0)
5	133.3 (*d*)	5.77 (1H, *m*)	133.5 (*d*)	5.77 (1H, *m*)	134.3 (*d*)	5.65 (1H, *dt*, 14.3, 6.5)
6	31.9 (*t*)	2.16 (2H, *t*, 2.9)	32.6 (*t*)	1.96 (2H, *q*-like, 6.2)	32.3 (*t*)	2.05 (2H, *m*)
7	32.1 (*t*)	2.04 (2H, *q*, 7.1)	32.4 (*t*)	2.11 (2H, *m*)	29.2–29.7 (*t*)	1.26 (2H, *br s*) ^b^
8	133.5 (*d*)	5.59 (1H, *dt*, 15.0, 6.0)	131.3 (*d*)	5.41 (1H, *m*)	29.2–29.7 (*t*)	1.26 (2H, *br s*) ^b^
9	130.0 (*d*)	5.98 (1H, *m*)	129.0 (*d*)	5.38 (1H, *m*)	29.2–29.7 (*t*)	1.26 (2H, *br s*) ^b^
10	131.2 (*d*)	6.00 (1H, *m*)	32.2 (*t*)	2.07 (2H, *m*)	29.2–29.7 (*t*)	1.26 (2H, *br s*) ^b^
11	130.7 (*d*)	5.52 (1H, *m*)	29.3–29.7 (*t*)	1.26 (2H, *br s*) ^b^	29.2–29.7 (*t*)	1.26 (2H, *br s*) ^b^
12	32.1 (*t*)	2.16 (2H, *t*, 2.9)	29.3–29.7 (*t*)	1.26 (2H, *br s*) ^b^	29.2–29.7 (*t*)	1.26 (2H, *br s*) ^b^
13	32.6 (*t*)	2.04 (2H, *q*, 7.1)	29.3–29.7 (*t*)	1.26 (2H, *br s*) ^b^	29.2–29.7 (*t*)	1.26 (2H, *br s*) ^b^
14	29.4 (*t*)	1.36 (2H, *m*)	29.3–29.7 (*t*)	1.26 (2H, *br s*) ^b^	29.2–29.7 (*t*)	1.26 (2H, *br s*) ^b^
15	29.2 (*t*)	1.36 (2H, *m*)	29.3–29.7 (*t*)	1.26 (2H, *br s*) ^b^	29.2–29.7 (*t*)	1.26 (2H, *br s*) ^b^
16	31.9 (*t*)	1.26 (2H, *br s*) ^b^	32.0 (*t*)	1.26 (2H, *br s*) ^b^	32.0 (*t*)	1.26 (2H, *br s*) ^b^
17	22.7 (*t*)	1.26 (2H, *br s*) ^b^	22.7 (*t*)	1.26 (2H, *br s*) ^b^	22.7 (*t*)	1.26 (2H, *br s*) ^b^
18	14.1 (*q*)	0.87 (3H, *t*, 7.1)	14.1 (*q*)	0.88(3H, *t*, 7.0)	14.1 (*q*)	0.88(3H, *t*, 7.1)
1′	175.1 (*s*)	-	173.0 (*s*)	-	173.0 (*s*)	-
2′	72.4 (*d*)	4.12 (1H, *dd*, 7.8, 3.4)	43.4 (*t*)	a 2.29 (1H, *dd*, 14.5, 9.5),	43.3 (*t*)	a 2.31 (1H, *m*), b 2.41 (1H, *m*)
				b 2.41 (1H, *d*, 14.3)		
3′	34.8 (*t*)	2.16 (2H, *t*, 2.9)	68.9 (*d*)	4.00 (1H, *m*)	68.9 (*d*)	4.00 (1H, *m*)
4′	31.8 (*t*)	1.26 (2H, *br s*) ^b^	37.2 (*t*)	a 1.43 (1H, *m*), b 1.51 (1H, *m*)	37.1 (*t*)	a 1.43 (1H, *m*), b 1.51 (1H, *m*)
5′	25.1 (*t*)	1.62 (2H, *m*)	25.6 (*t*)	a 1.26 (1H, *br s*) ^b^, b 1.43 (1H, *m*)	25.6 (*t*)	a 1.30 (1H, *m*), b 1.43 (1H, *m*)
6′~13′	29.1–29.7 (*t*)	1.26 (16H, *br s*) ^b^	29.3–29.7 (*t*)	1.26 (16H, *br s*) ^b^	29.2–29.7 (*t*)	1.26 (16H, *br s*) ^b^
14′	29.1–29.7 (*t*)	1.26 (2H, *br s*) ^b^	32.0 (*t*)	1.26 (2H, *br s*) ^b^	32.0 (*t*)	1.26 (2H, *br s*) ^b^
15′	29.1–29.7 (*t*)	1.26 (2H, *br s*) ^b^	22.7 (*t*)	1.26 (2H, *br s*) ^b^	22.7 (*t*)	1.26 (2H, *br s*) ^b^
16′	31.9 (*t*)	1.26 (2H, *br s*) ^b^	14.1 (*q*)	0.88 (3H, *t*, 7.0)	14.1 (*q*)	0.88 (3H, *t*, 7.1)
17′	22.7 (*t*)	1.26 (2H, *br s*) ^b^	-	-	-	-
18′	14.1 (*q*)	0.87 (3H, *t*, 7.1)	-	-	-	-
NH	-	7.20 (1H, *d*, 7.9)	-	6.76 (1H, *d*, 7.8)	-	6.77 (1H, *d*, 7.8)

^a^ Assignments aided by DEPT, ^1^H-^1^H COSY, HSQC, HMBC and NOESY experiments; ^b^ overlapped with other signals.

**Figure 6:**
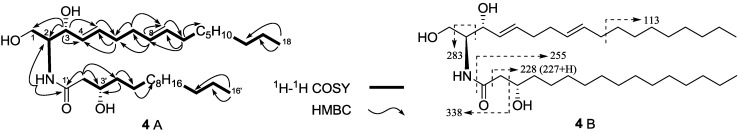
Key ^1^H–^1^H COSY and HMBC correlations (**A**), and negative ESI-MS fragments (**B**) of compound **4**.

The molecular formula of neritinaceramide D (**4**) was assigned as C_34_H_65_NO_4_ on the basis of HR-ESI-MS pseudomolecular ion peak at *m/z* 574.4815 [M + Na]^+^ (calcd. for C_34_H_65_NO_4_Na, 574.4811) and the results of ^1^H and ^13^C NMR spectral interpretations (Table 2). Again, the ^1^H- and ^13^C-NMR spectra of **4** were found to be nearly identical to those of **1** except for the missing of two olefinic carbons at C-10 and C-11 in the LCB. The positions of the four reminder olefinic carbons *δ* 133.5 (C-5), 131.3 (C-8), 129.0 (C-9) and 128.9 (C-4) were determined to be C-4 and C-8 mainly by ^1^H-^1^H COSY correlations from H-3 (*δ* 4.28) to H-4 (*δ* 5.51), and in turn, from H-4 to H-5 (*δ* 5.77), H-5 to H_2_-6 (*δ* 1.96), H_2_-6 to H_2_-7 (*δ* 2.11), H_2_-7 to H-8 (*δ* 5.41), H-8 to H-9 (*δ* 5.38) and H-9 to H_2_-10 (*δ* 2.07), and further confirmed by HMBC correlations from H-3 to C-4 and C-5, H-5 to C-6 (*δ* 32.6), H_2_-6 to C-4 and C-5, H-8 to C-6, C-7 (*δ* 32.4) and C-9, and H-9 to C-10 (*δ* 32.2) (Figure 6A). Ceramide **4** possessed the similar negative ESI-MS fragment ions as compared with **1**, while the fragment ion *m/z* 338 of **4** replaced the fragment ion *m/z* 336 of **1** (Figure 6B). This also confirmed the missing of two olefinic carbons in **4**, and further deduced that **1** and **4** possessed the same carbon number in their long aliphatic chain. The stereochemistry of **4** was determined by the same method with those of **1**. Finally, neritinaceramide D (**4**) was elucidated as (2*S*,3*R*,3′*S*,4*E*,8*E*)-2-(hexadecanoylamino)-4,8-octadecadiene-l,3,3′-triol.

**Figure 7:**
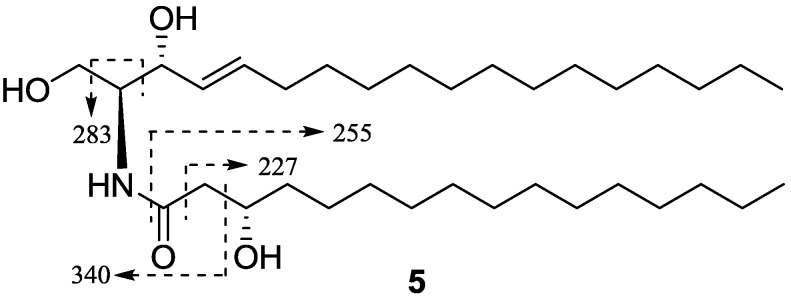
Key negative ESI-MS fragments of compound **5**.

Neritinaceramide E (**5**) was obtained as a white amorphous powder with a molecular formula of C_34_H_67_NO_3_, determined by positive HR-ESI-MS *m/z* 576.4967 [M + Na]^+^ (calcd. for C_34_H_67_NO_3_Na, 576.4968), and the negative ESI-MS *m/z* 552 [M − H]^−^, with the help of NMR data. Comparison of the NMR spectra of **5** with **4** indicated that they possessed the same ceramide skeleton, except for the absence of a double bond between C-8 and C-9 of **5**. The fragment ions of **5** were almost identical with those of **4** in the negative ESI-MS, but the fragmet ion *m/z* 338 in **4** was replaced by fragmet ion *m/z* 340 in **5** (Figure 7). This indicated that the numbers of carbons of the FAB and LCB were also 16 and 18, respectively, and allowed the assignment of structure **5** to be (2*S*,3*R*,3′*S*,4*E*)-2-(hexadecanoylamino)-4-octadecene-l,3,3′-triol.

The remainder six known ceramides were established as (2*S*,3*R*,2′*R*,4*E*)-2-(hexadecanoylamino)-4-octadecene-l,3,2′-triol (**6**) [28], (2*S*,3*R*,2′*R*,4*E*,8*E*)-2-(hexadecanoylamino)-4,8-octadecadiene-l,3,2′-triol (**7**) [29], (2*S*,3*R*,4*E*)-2-(hexadecanoylamino)-4-octadecene-l,3-diol (**8**) [30], (2*S*,3*R*)-2-(hexadecanoylamino)-4-octadecane-l,3-diol (**9**) [14], (2*S*,3*R*,4*E*)-2-(hexadecanoylamino)-4-nonadecene-l,3-diol (**10**) [14], and (2*S*,3*R*,4*E*)-2-(hexadecanoylamino)-4-eicosene-l,3-diol (**11**) [14], and a known nucleoside was elucidated as adenine riboside (**14**) [31], by comparing their spectroscopic data with those published in the literatures. The ^1^H- and ^13^C-NMR spectra of **12** and **13** showed a resemblance to those identified ceramides, since they shared the similar long chain and oxygen-bearing signals. However, the structural type of **12** and **13** was determined as alkyl glycerylether, since they lack a carbonyl carbon signal at *δ* 173.0–175.1 in the ^13^C-NMR spectra, and the loss of NH signal at *δ* 6.65–8.34 in the ^1^H-NMR spectra. The ^13^C-NMR and DEPT spectra of **12** showed an oxygenated methine at *δ* 64.5 (C-1) and three oxygenated methylenes at *δ* 64.5 (C-1), 72.0 (C-3) and 72.7 (C-16′), indicating the terminal substituted alkyl glycerylether for **12**. The pseudomolecular ion peak at *m/z* 339 [M + Na]^+^ in the positive ESI-MS, along with the analysis of NMR spectral data enabled the determination of the molecular formula of **12** as C_19_H_40_O_3_, and 16 carbons for the alkyl long chain accordingly. Thus, the structure of **12** was determined as 1-*O*-palmityl glycerin ether [32]. Similarly, alkyl glycerylether **13**, also known as batiolum, was elucidated as 1-*O*-octadecyl glycerin ether based on the pseudomolecular ion peak at *m/z* 367 [M + Na]^+^ in the positive ESI-MS, with the help of NMR spectral data [33]. Ceramides **9**–**11**, alkyl glycerylethers **12** and **13**, and nucleoside (**14**) were isolated from marine bryozoans for the first time. Besides, batiolum (**13**) has been developed as medicine to increase the level of leukocyte [34].

The new ceramides **1**–**5** were evaluated for their cytotoxicity against human tumor cell lines HepG2, NCI-H460 and SGC7901 *in vitro* with a method of MTT assay. The results of their cytotoxicity were shown in Table 3. Ceramides **1**–**5** exhibited selective cytotoxicity against HepG2 and SGC7901 cells with IC_50_ values range from 47.3 μM to 58.1 μM, while showed relative weak activity to NCI-H460 cell line with IC_50_ values more than 84.4 μM. The above information suggested a good selectivity of those ceramides against different tumor cell lines. Besides, ceramides **2** and **3** characterized at a 2′*R* hydroxyl group, showed more activity than ceramides **1**, **4** and **5** with a 3′*S* hydroxyl group. This indicated that the 2′*R* hydroxyl group in FAB is important for their cytotoxicity [35]. However, all of the test ceramides appeared lower cytotoxicity compared with corresponding glycosylated-ceramides, cerebrosides, which suggested that the sugar moiety is critical to cytotoxic activity [36]. In fact, multiple related studies indicated that the structure-activity relationships (SAR) about the antitumor activity of ceramides and cerebrosides were rendered by the presence of the *trans* double bond between C-4 and C-5 in the vicinity of their polar head [37,38], the category of the sugar moieties at C-1 in the LCB [36,39], and the additional hydroxyl group at position C-2′ or C-4 [3,35]. The SAR studies of those ceramides provided potential chemical modification values to search for approach to expand their biological properties.

**Table 3:** Cytotoxicities of compounds **1**–**5** against three tumor cell lines *in vitro* (IC_50_, μM) ^a^.

Compound	HepG2	NCI-H460	SGC7901
**1**	52.1	>100	57.7
**2**	47.3	84.4	52.2
**3**	47.8	85.1	52.9
**4**	53.7	>100	57.9
**5**	54.6	>100	58.1
Adriamycin ^b^	0.14	0.13	0.17

^a^ IC_50_ values are means from three independent experiments in which each compound concentration was tested in three replicate wells; ^b^ Adriamycin as positive control.

Among the marine invertebrates, the primary ceramides and cerebrosides were often found as those derived from the doubly unsaturated LCB with 4*E*-sphinganine and 4*E*,8*E*-sphingadiene [19], as well as from the saturated phytosphingosine, 4-hydroxysphingosine [40]. To date, the 4*E*,8*E*,10*E*-sphingatriene LCB in ceramides and cerebrosides were rare encountered in natural, and just isolated from the star fish *Ophidiaster ophidiamus* [2], ascidian *Phallusia fumigate* [41] and gorgonian *Acabaria undulate* [19]. Ceramides (**1**−**3**) characterized at a 4*E*,8*E*,10*E*-sphingatriene LCB were isolated from marine bryozoans for the first time. Interestingly, to the best of our knowledge, naturally occurring FAB in ceramides included no hydroxyl substituted form [19] and 2′(*R*)-hydroxyl substituted form [3]. The 3′(*S*)-hydroxyl substituted FAB in compounds **1**, **4** and **5** was a novel structural feature in ceramides. Thus, the discovery of those five new ceramides, neritinaceramides A−E (**1**−**5**), from *Bugula neritina* was a typical example to illustrate the chemical diversity of the title species.

## 3. Experimental Section

### 3.1. General Experimental Procedures

Melting points were determined on an XT5-XMT apparatus and uncorrected. Specific rotation [α]_D_ was obtained on a Perkin-Elmer 343 polarimeter. 1D and 2D NMR spectral experiments were measured in CDCl_3_ or pyridine-*d*_5_ on a Bruker AVANCE-500 spectrometer with TMS as an internal standard. Chemical shifts (*δ*) were expressed in ppm and coupling constants (*J*) in Hz. EI-MS spectra were obtained on a Finnigan MAT 212 mass spectrometer. ESI-MS and HR-ESI-MS spectra were carried out on a Micromass Quattro Micro mass spectrometer. Column chromatography (CC) was performed on silica gel H (10−40 μm, Qingdao Marine Chemical Inc., Qingdao, China) and Sephadex LH-20 (GE-Healthcare, Sweden). HPLC was carried out on a Dionex P680 liquid chromatograph equipped with a UV 170 UV/Vis detector at 206 nm using a YMC-Pack R & D ODS-A column (250 × 20 mm i.d., 5 μm, YMC, Kyoto, Japan) for semi-preparation and a Thermo ODS-2 column (250 × 4.6 mm i.d., 5 μm, Thermo Hypersi-Keystone Inc., Bellefonte, USA) for analysis. TLC detection was achieved by spraying the silica gel plates (Qingdao Marine Chemical Inc., Qingdao, China) with 10% H_2_SO_4_ in EtOH, followed by heating for 3 min.

### 3.2. Animal Material

Marine bryozoan *Bugula neritina* was collected in March 2008 in Daya Bay, Shenzhen, China. The specimen identification was conducted by Prof. H.-W. Lin. A voucher specimen numbered 20080312, was deposited in the Herbarium of Pharmacy, Changzheng Hospital, Second Military Medical University, Shanghai, China.

### 3.3. Extraction and Isolation

The marine bryozoan *B. neritina* (about 100 kg, dry weight) was extracted with 95% EtOH (4 × 500 L) at room temperature with each extraction taking one week. The concentrated EtOH extract (2.0 kg) was suspended in water, and then partitioned successively with EtOAc to obtained EtOAc extract (600 g). One third of the EtOAc extract was subjected to CC over silica gel (1200 g, 10−40 μm) eluting with the CHCl_3_-MeOH (100:1, 50:1, 20:1, 10:1, 1:1, 0:1) gradient to afford nine major fractions (A−I) based on TLC analysis. Fr. E (15.0 g) was subject to CC on silica gel (150 g, 10−40 μm) eluting with petroleum ether-EtOAc (10:1 to 1:1) gradient to give four major fractions (Fr. E1−E4). The Fr. E3 (2.5 g) was recrystallisated with CH_3_COCH_3_ to obtained a crude white powder (900 mg), and then further purified by semi-preparative reverse HPLC with MeOH-H_2_O (97:3) as the mobile phase at a flow rate of 8.0 mL/min to afford five new ceramides **1** (3.7 mg, *t*_R_ = 32.9 min), **2** (11.5 mg, *t*_R_ = 39.9 min), **3** (19.7 mg, *t*_R_ = 38.3 min), **4** (50.3 mg, *t*_R_ = 45.5 min) and **5** (8.5 mg, *t*_R_ = 57.9 min). Fr. E4 (1.3 g) was eluted with CHCl_3_-MeOH (1:1) on Sephadex LH-20 to remove pigments, and then further purified by semi-preparative reverse HPLC using MeOH-H_2_O (97:3) as mobile phase at a flow rate of 8.0 mL/min to afford ceramides **6** (11.4 mg, *t*_R_ = 41.5 min) and **7** (8.7 mg, *t*_R_ = 43.7 min). Similarly, the remainder four known ceramides **8** (34.0 mg, *t*_R_ = 65.4 min), **9** (14.4 mg, *t*_R_ = 75.3 min), **10** (10.0 mg, *t*_R_ = 63.2 min) and **11** (12.6 mg, *t*_R_ = 94.4 min) were obtained from Fr. E2 (176.5 mg) by the same method with Fr. E4. The two known alkyl glycerylethers **12** (13.0 mg, *t*_R_ = 37.2 min) and **13** (18.7 mg, *t*_R_ = 39.5 min) were isolated from Fr. D via CC on silica gel column with 5:1 ether-EtOAc as eluting solvent, and finally further purified by semi-preparative reverse HPLC using MeOH-H_2_O (97:3) as mobile phase at a flow rate of 8.0 mL/min. The known nucleoside **14** (9.0 mg, *t*_R_ = 24.7 min) was purfied from Fr. H via CC over repeated sephadex LH-20 with CHCl_3_-MeOH (1:1) as eluting solvent, and then further purified by semi-preparative HPLC with MeOH-H_2_O (90:10) as mobile phase at a flow rate of 7.0 mL/min.

#### 3.3.1. Neritinaceramide A (**1**)

White amorphous powder, mp 109−110 °C; [α]^20^_D_ −4.5° (*c* 0.05, CHCl_3_); ^1^H-NMR (500 MHz, CDCl_3_) and ^13^C-NMR (125 MHz, CDCl_3_) data see Table 1; Positive ESI-MS *m*/*z* 572 [M + Na]^+^, 437, 409, 330, 312, 303, 284, 275, 256; Negative ESI-MS *m*/*z* 548 [M − H]^−^, 584 [M + Cl]^−^, 336, 325, 311, 297, 283, 255, 227, 165, 113; Positive HR-ESI-MS *m*/*z* 572.4658 [M + Na]^+^ (calcd. for C_34_H_63_NO_4_Na, 572.4655).

#### 3.3.2. Neritinaceramide B (**2**)

White amorphous powder, mp 111−112 °C; [α]^20^_D_ +9.8° (*c* 0.14, CHCl_3_); ^1^H-NMR (500 MHz, C_5_D_5_N) and ^13^C-NMR (125 MHz, C_5_D_5_N) data see Table 1; Positive ESI-MS *m*/*z* 572 [M + Na]^+^, 437, 409, 312, 284, 274, 256, 166; Negative ESI-MS *m*/*z* 548 [M − H]^−^, 584 [M + Cl]^−^, 283, 255, 227, 113; Positive HR-ESI-MS *m*/*z* 572.4652 [M + Na]^+^ (calcd. for C_34_H_63_NO_4_Na, 572.4655).

#### 3.3.3. Neritinaceramide C (**3**)

White amorphous powder, mp 105−106 °C; [α]^20^_D_ +10.1° (*c* 0.10, CHCl_3_); ^1^H-NMR (500 MHz, CDCl_3_) and ^13^C-NMR (125 MHz, CDCl_3_) data see Table 2; Positive ESI-MS *m*/*z* 600 [M + Na]^+^, 437, 409, 330, 311, 283, 274, 264, 256, 219, 166; Negative ESI-MS (−) *m*/*z* 576 [M − H]^−^, 340, 324, 312, 298, 283, 271, 255, 253, 237, 225, 113.

#### 3.3.4. Neritinaceramide D (**4**)

White amorphous powder, mp 104−105 °C; [α]^20^_D_ −4.3° (*c* 0.15, CHCl_3_); ^1^H-NMR (500 MHz, CDCl_3_) and ^13^C-NMR (125 MHz, CDCl_3_) data see Table 2; Positive ESI-MS *m*/*z* 574 [M + Na]^+^, 1125 [2M + Na]^+^, 437, 409, 274, 262, 256; Negative ESI-MS *m*/*z* 550 [M − H]^−^, 586 [M + Cl]^−^, 338, 283, 255, 228, 113; Positive HR-ESI-MS *m*/*z* 574.4815 [M + Na]^+^ (calcd. for C_34_H_65_NO_4_Na, 574.4811).

#### 3.3.5. Neritinaceramide E (**5**)

White amorphous powder, mp 101−102 °C; [α]^20^_D_ −4.1° (*c* 0.10, CHCl_3_); ^1^H-NMR (500 MHz, CDCl_3_) and ^13^C-NMR (125 MHz, CDCl_3_) data see Table 2; Positive ESI-MS *m*/*z* 576 [M + Na]^+^, 437, 409, 311, 300, 283, 274, 264, 256, 228; Negative ESI-MS *m*/*z* 552 [M − H]^−^, 588 [M + Cl]^−^, 340, 283, 255, 227, 113; Positive HR-ESI-MS *m*/*z* 576.4967 [M + Na]^+^ (calcd. for C_34_H_67_NO_3_Na, 576.4968).

The copies of the original spectra of the new compounds **1**−**5** are available in Supplementary Information.

#### 3.3.6. 1-*O*-Palmityl Glycerin Ether (**12**)

White amorphous powder, mp 60–62 °C. ^1^H-NMR (500 MHz, CDCl_3_) *δ* 0.88 (3H, *t*, *J* = 6.5 Hz, H_3_-1′), 1.26 (26H, *br s*, H_2_-2′ to H_2_-14′), 1.58 (2H, *t*, *J* = 7.0 Hz, H_2_-15′), 3.52 (4H, *m*, H_2_-3 and H_2_-16′), 3.65 (1H, *dd*, *J* = 11.0, 5.0 Hz, H_a_-1), 3.72 (1H, *dd*, *J* = 11.5, 3.5 Hz, H_b_-1), 3.86 (1H, *m*, H-2); ^13^C-NMR (125 MHz, CDCl_3_) *δ* 64.5 (C-1), 70.6 (C-2), 72.0 (C-3), 14.3 (C-1′), 23.1 (C-2′), 32.1 (C-3′), 29.1-29.8 (C-4′~13′), 26.2 (C-14′), 29.8 (C-15′), 72.7 (C-16′); Positive ESI-MS *m/z* 339 [M + Na]^+^, 655 [2M + Na]+; Negative ESI-MS *m/z* 631 [2M − H]^−^, 351 [M + Cl]^−^, 361 [M + COOH]^−^.

#### 3.3.7. 1-*O*-Octadecyl Glycerin Ether (**13**)

White amorphous powder, mp 62–63 °C. ^1^H-NMR (500 MHz, CDCl_3_) *δ* 0.86 (3H, *t*, *J* = 7.0 Hz, H_3_-1′), 1.27 (30H, *br s*, H_2_-2′ to H_2_-16′), 1.57 (2H, *t*, *J* = 7.0 Hz, H_2_-17′), 3.49 (4H, *m*, H_2_-3 and H_2_-18′), 3.63 (1H, *dd*, *J* = 11.5, 5.5 Hz, H_a_-1), 3.72 (1H, *dd*, *J* = 11.5, 4.0 Hz, H_b_-1), 3.86 (1H, *m*, H-2); ^13^C-NMR (125 MHz, CDCl_3_) *δ* 64.4 (C-1), 70.6 (C-2), 72.0 (C-3), 14.3 (C-1′), 22.8 (C-2′), 32.1 (C-3′), 29.5-29.9 (C-4′~15′), 26.2 (C-16′), 29.9 (C-17′), 72.6 (C-18′); Positive ESI-MS *m/z* 367 [M + Na]^+^, 711 [2M + Na]^+^.

### 3.4. Methanolysis of Ceramides 1−5

Each ceramide (about 2 mg) was dissolved in 5% HCl-MeOH (2 mL) and refluxed for 10 h at 80 °C. The reaction mixture was extracted with *n*-hexane for three times. The *n*-hexane layer was washed with H_2_O and concentrated *in vacuo* to yield the corresponding fatty acid methyl ester (FAME **1*−*5**) [14]. The remainder H_2_O layer was neutralized with NH_4_OH, and then further concentrated *in vacuo* to yield the corresponding long chain base (LCB **1**–**5**) with specific rotation [α]^20^_D_ about −2.8° (*c* 0.10, CHCl_3_).

(*S*)-Methyl 3-hydroxylhexadecanoate (FAME **1**, **4** and **5**): [α]^20^_D_ +12.5° (*c* 0.11, CHCl_3_); EI-MS *m*/*z*286 [M]^+^ (4), 268 [M *−* H_2_O]^+^ (9), 236 (6), 211 (4), 194 (7), 111 (9), 103 (100), 83 (10), 74 (22), 55 (19).

(*R*)-Methyl 2-hydroxylhexadecanoate (FAME **2)**: [α]^20^_D_ *−* 6.7° (*c* 0.15, CHCl_3_); EI-MS *m*/*z* 286 [M]^+^ (9), 254 [M *−* OCH_3_ *−*H]^+^ (4), 227 (67), 208 (6), 145 (13), 111 (50), 103 (21), 97 (96), 83 (100).

(*R*)-Methyl 2-hydroxyloctadecanoate (FAME **3)**: [α]^20^_D_ *−* 6.5° (*c* 0.10, CHCl_3_); EI-MS *m*/*z* 314 [M]^+^ (26), 296 [M *−* H_2_O]^+^ (4), 282 [M *−* OCH_3_ *−* H]^+^ (6), 269 (5), 255 (76), 236 (6), 145 (14), 111 (52), 103 (19), 97 (93), 83 (85), 57 (100).

### 3.5. MTT Cytotoxicity Assays

Neritinaceramides A−E (**1−5**) were evaluated for their cytotoxic activity against human hepatoma HepG2, pulmonary carcinoma NCI-H460 and gastric carcinoma SGC7901 tumor cells by microculture tetrazolium (MTT) assay. The cells were obtained from American Type Culture Collection (ATCC), and were seeded to RPMI-1640 medium with 10% fetal bovine serum and 100 U/mL benzyl penicillin-streptomycin solution at 37 °C in a humidified atmosphere containing in 5% CO_2_/air for 24 h. Then the test samples were added and incubated at 37 °C for another 72 h. The detailed experiments procedure has been described in our previous published literature [16]. The inhibition was expressed as IC_50_ value with adriamycin as positive control.

## 4. Conclusions

The present chemical study of the marine bryozoan *B. neritina* resulted in the isolation and characterization of five new ceramides, neritinaceramides A−E (**1**−**5**), six known ceramides (**6**–**11**), two known alkyl glycerylethers (**12** and **13**) and a known nucleoside (**14**). The characteristic C-3′*S* hydroxyl group in the fatty acid moiety in compounds **1**, **4** and **5**, was a novel structure feature in ceramides. The rare 4*E*,8*E*,10*E*-triene structure in the sphingoid base of compounds **1**–**3**, was found from marine bryozoans for the first time. Bioacitve studies indicated that those new ceramides (**1**−**5**) exhibited selective cytotoxicity against HepG2 and SGC7901 cells with IC_50_ values range from 47.3 μM to 58.1 μM. These chemical and cytotoxic studies on the new neritinaceramides A–E (**1**–**5**) added to the chemical diversity of *B*. *neritina* and expanded our knowledge of the chemical modifications and biological activity of ceramides.

## Supplementary Files

Supplementary File 1 Supplementary Information (PDF, 1840 KB)
