# Supplementary material for: Neritinaceramides A–E, New Ceramides from the Marine Bryozoan Bugula neritina Inhabiting South China Sea and Their Cytotoxicity
Source: Mar Drugs. 2014 Apr 2;12(4):1987–2003. doi: 10.3390/md12041987 (PMC4012440; doi:10.3390/md12041987)
Supplement: Supplementary File 1 — Supplementary Information (PDF, 1840 KB) [file marinedrugs-12-01987-s001.pdf]

Supplementary Information

Figure S1. HR-ESI-MS (positive) spectrum of compound **1**.
Figure S2. ESI-MS (positive and negative) spectrum of compound **1**.
Figure S3. ESI-MS (positive and negative) fragments spectrum of compound **1**.
Figure S4. ^{1}H-NMR spectrum of compound **1**.
Figure S5. ^{13}C-NMR spectrum of compound **1**.
Figure S6. DEPT spectrum of compound **1**.
Figure S7. HSQC spectrum of compound **1**.
Figure S8. HMBC spectrum of compound **1**.
Figure S9. ^{1}H-^{1}H COSY spectrum of compound **1**.
Figure S10. NOESY spectrum of compound **1**.
Figure S11. EI-MS data of FAME **1**.
Figure S12. HR-ESI-MS (positive) spectrum of compound **2**.
Figure S13. ESI-MS (positive and negative) spectrum of compound **2**.
Figure S14. ESI-MS (positive and negative) fragments spectrum of compound **2**.
Figure S15. ^{1}H-NMR spectrum of compound **2**.
Figure S16. ^{13}C-NMR spectrum of compound **2**.
Figure S17. DEPT spectrum of compound **2**.
Figure S18. HSQC spectrum of compound **2**.
Figure S19. HMBC spectrum of compound **2**.
Figure S20. ^{1}H-^{1}H COSY spectrum of compound **2**.
Figure S21. NOESY spectrum of compound **2**.
Figure S22. EI-MS data of FAME **2**.
Figure S23. ESI-MS (positive and negative) spectrum of compound **3**.
Figure S24. ESI-MS (positive and negative) fragments spectrum of compound **3**.
Figure S25. ^{1}H-NMR spectrum of compound **3**.
Figure S26. ^{13}C-NMR spectrum of compound **3**.
Figure S27. DEPT spectrum of compound **3**.
Figure S28. EI-MS data of FAME **3**.
Figure S29. HR-ESI-MS (positive) spectrum of compound **4**.
Figure S30. ESI-MS (positive and negative) spectrum of compound **4**.
Figure S31. ESI-MS (positive and negative) fragments spectrum of compound **4**.
Figure S32. ^{1}H-NMR spectrum of compound **4**.
Figure S33. ^{13}C-NMR spectrum of compound **4**.
Figure S34. DEPT spectrum of compound **4**.
Figure S35. HR-ESI-MS (positive) spectrum of compound **5**.
Figure S36. ESI-MS (positive and negative) spectrum of compound **5**.
Figure S37. ESI-MS (positive and negative) fragments spectrum of compound **5**.
Figure S38. ^{1}H-NMR spectrum of compound **5**.
Figure S39. ^{13}C-NMR spectrum of compound **5**.
Figure S40. DEPT spectrum of compound **5**.

Figure S1. HR-ESI-MS (positive) spectrum of compound **1**.

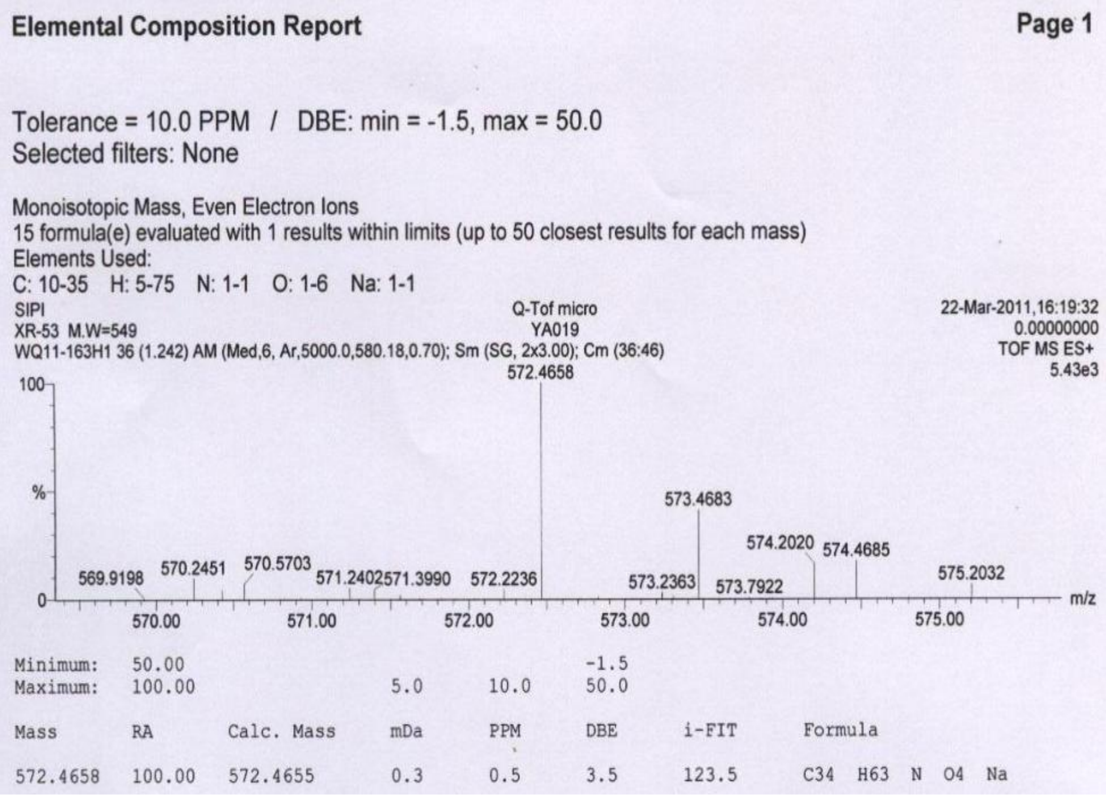

Elemental Composition Report Page 1

Tolerance = 10.0 PPM / DBE: min = -1.5, max = 50.0
Selected filters: None

Monoisotopic Mass, Even Electron Ions
15 formula(e) evaluated with 1 results within limits (up to 50 closest results for each mass)
Elements Used:
C: 10-35 H: 5-75 N: 1-1 O: 1-6 Na: 1-1

Minimum:	50.00				-1.5		
Maximum:	100.00		5.0	10.0	50.0		

Mass	RA	Calc. Mass	mDa	PPM	DBE	i-FIT	Formula
572.4658	100.00	572.4655	0.3	0.5	3.5	123.5	C34 H63 N O4 Na

Figure S2. ESI-MS (positive and negative) spectrum of compound **1**.

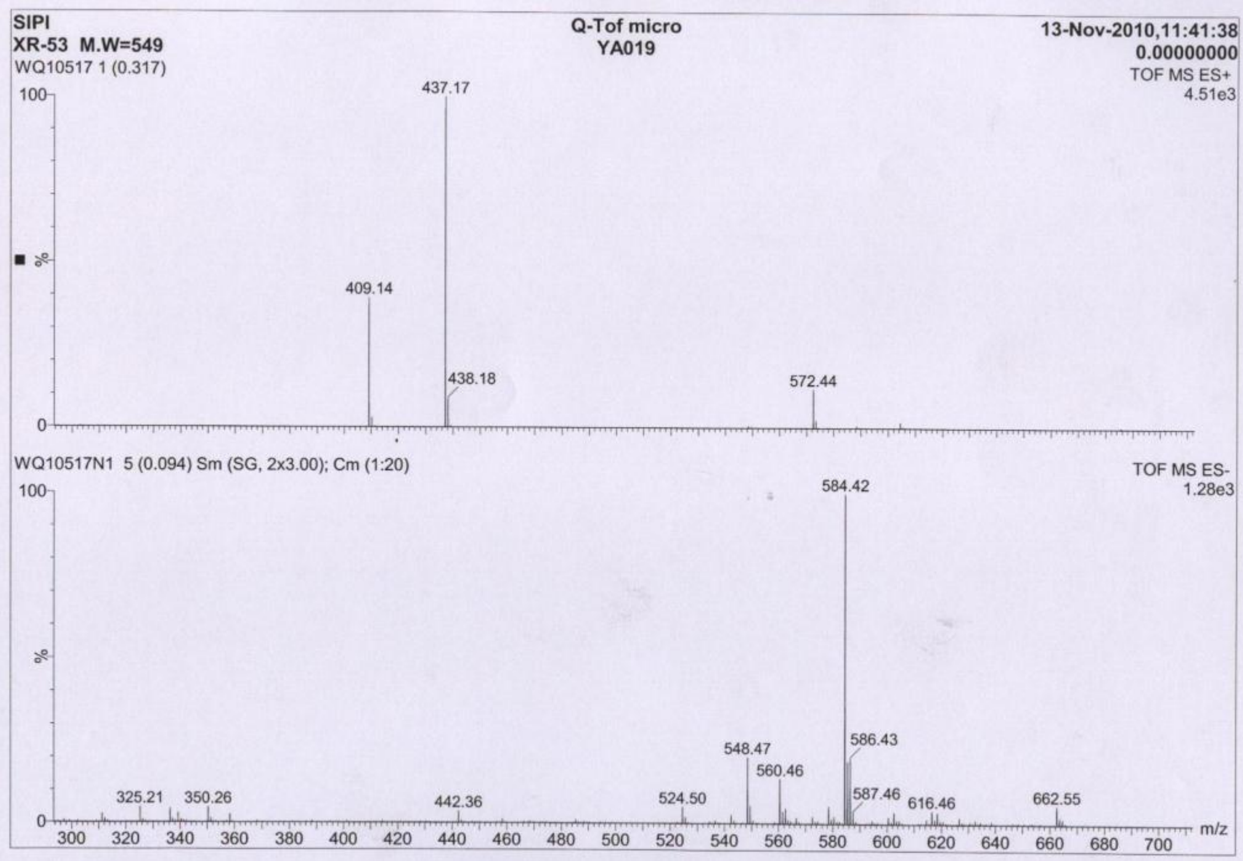

Figure S3. ESI-MS (positive and negative) fragments spectrum of compound **1**.

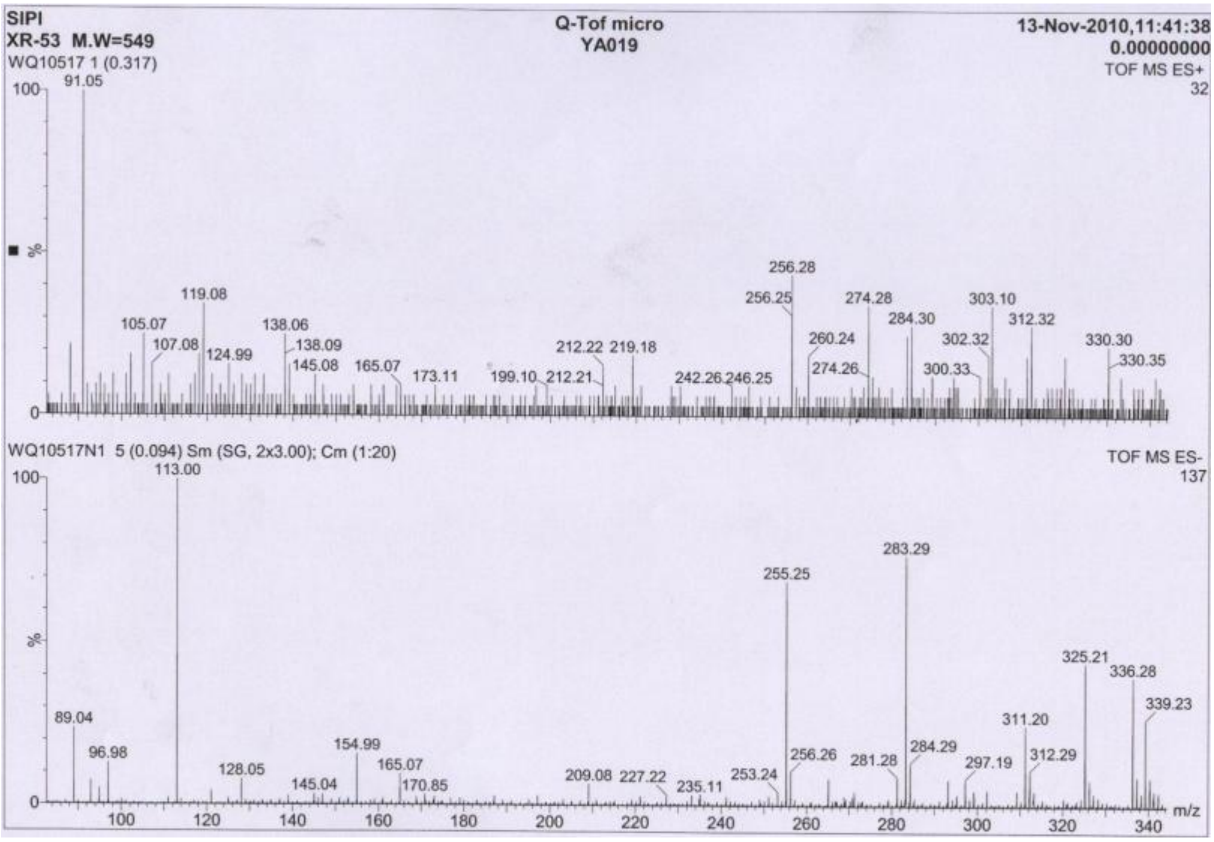

Figure S4. ^{1}H-NMR spectrum of compound **1**.

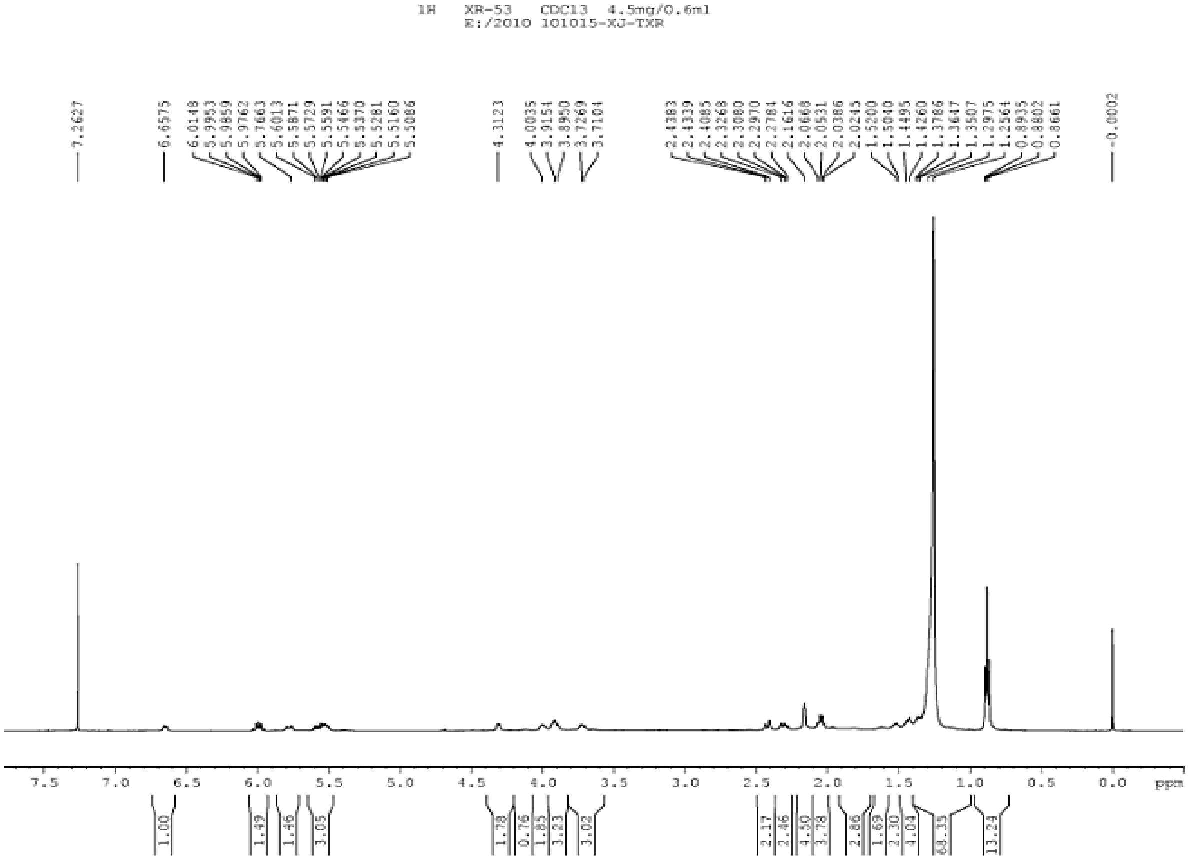

Figure S5. ^{13}C-NMR spectrum of compound **1**.

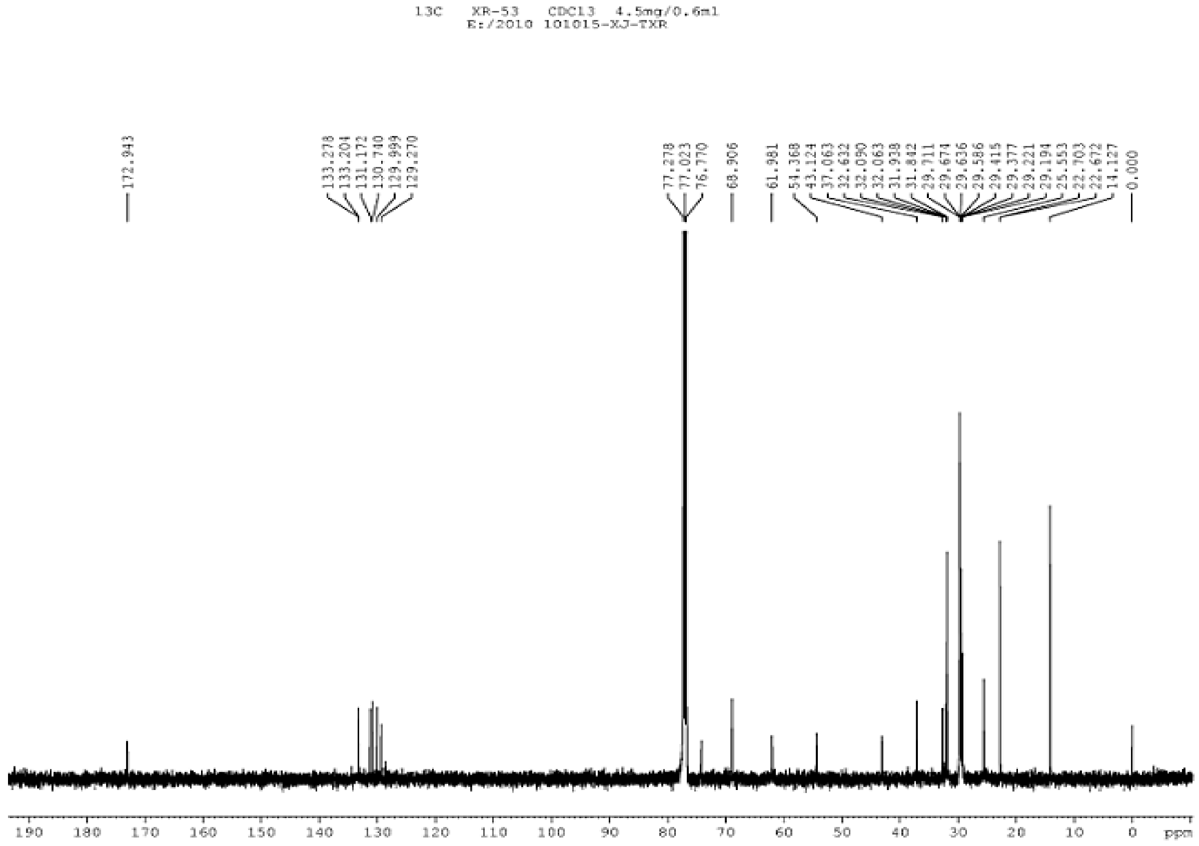

Figure S6. DEPT spectrum of compound **1**.

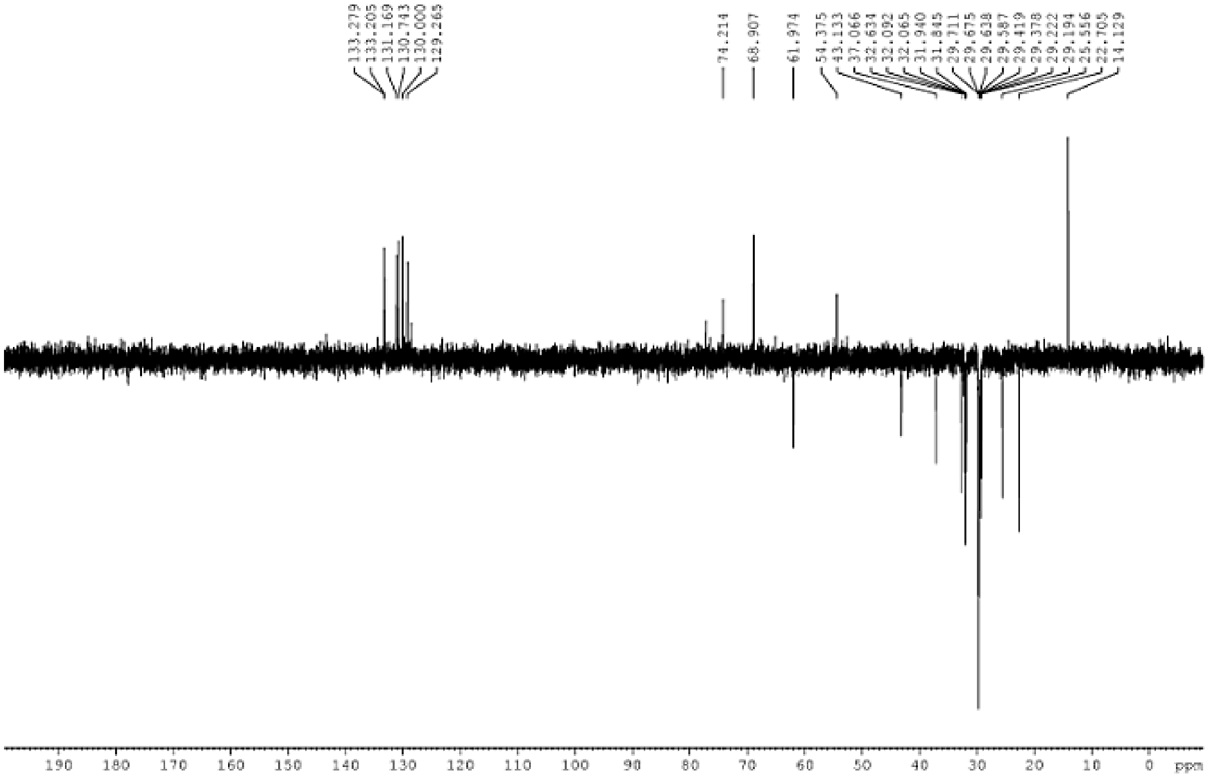

Figure S7. HSQC spectrum of compound **1**.

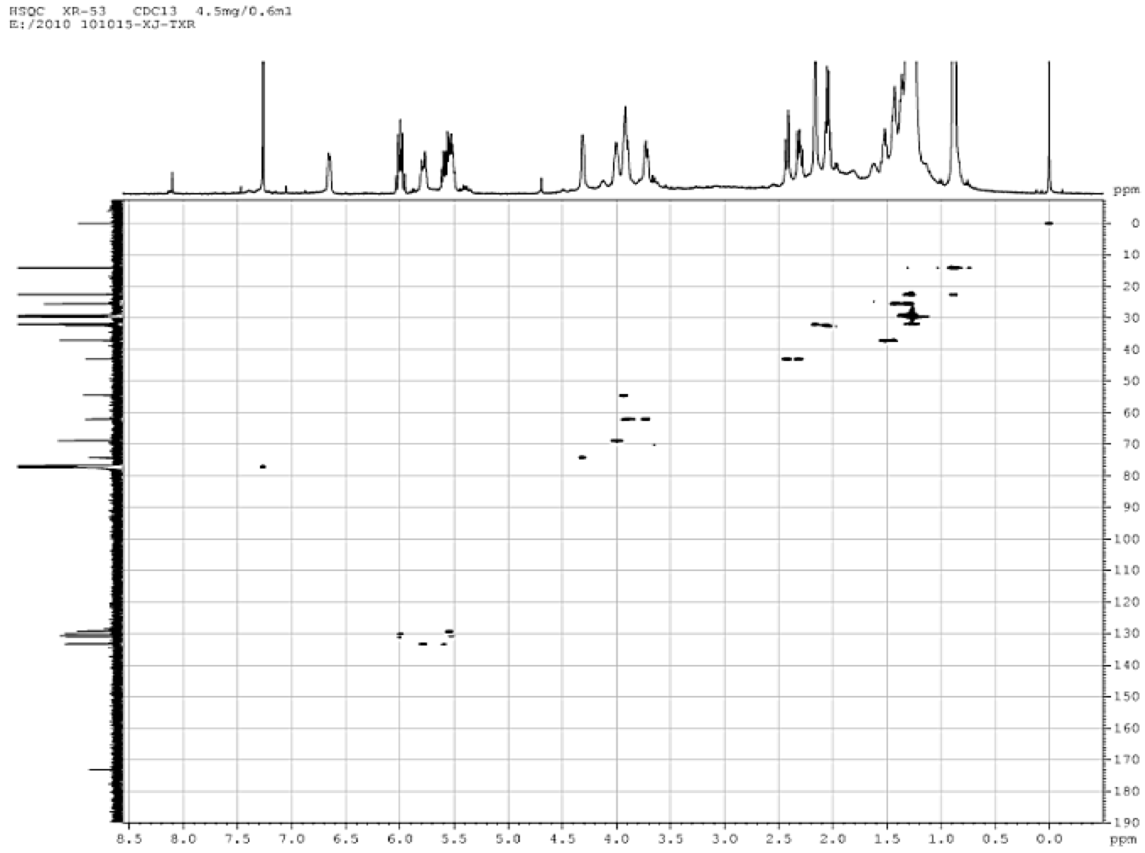

Figure S8. HMBC spectrum of compound **1**.

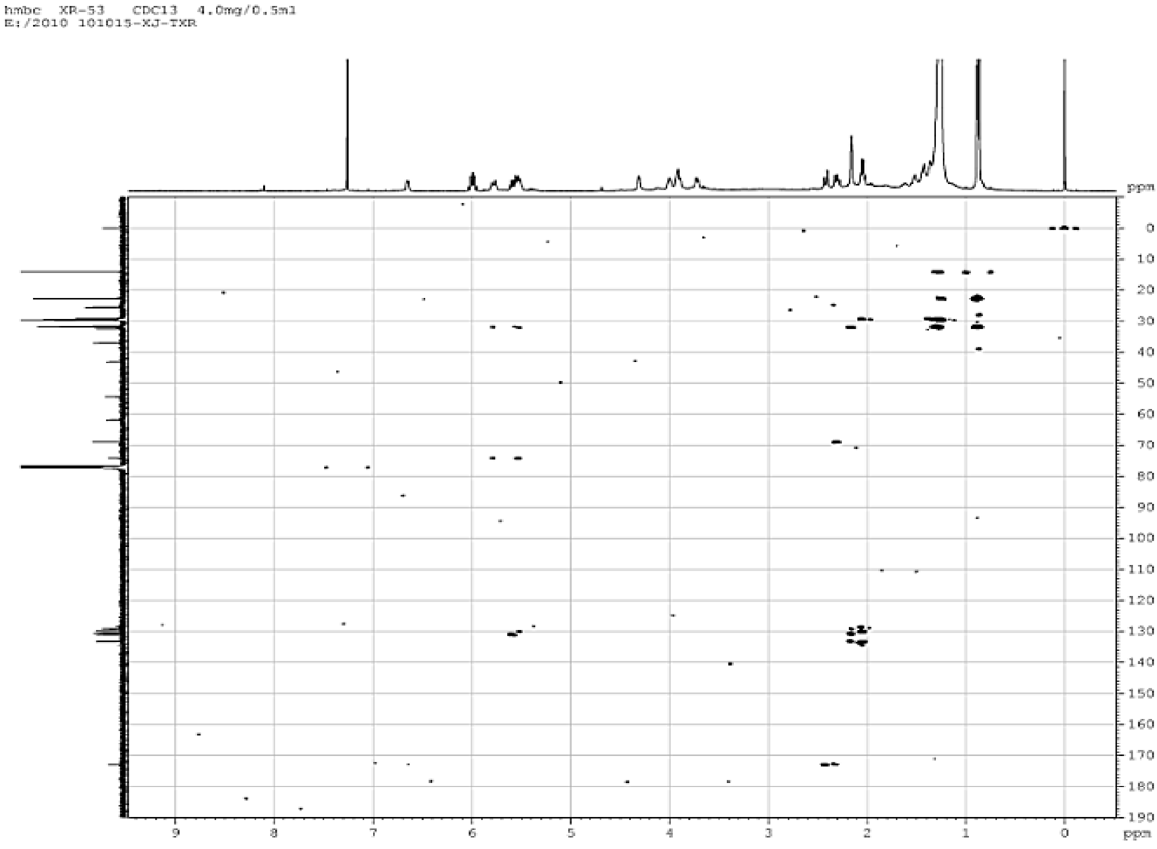

Figure S9. ^{1}H-^{1}H COSY spectrum of compound **1**.

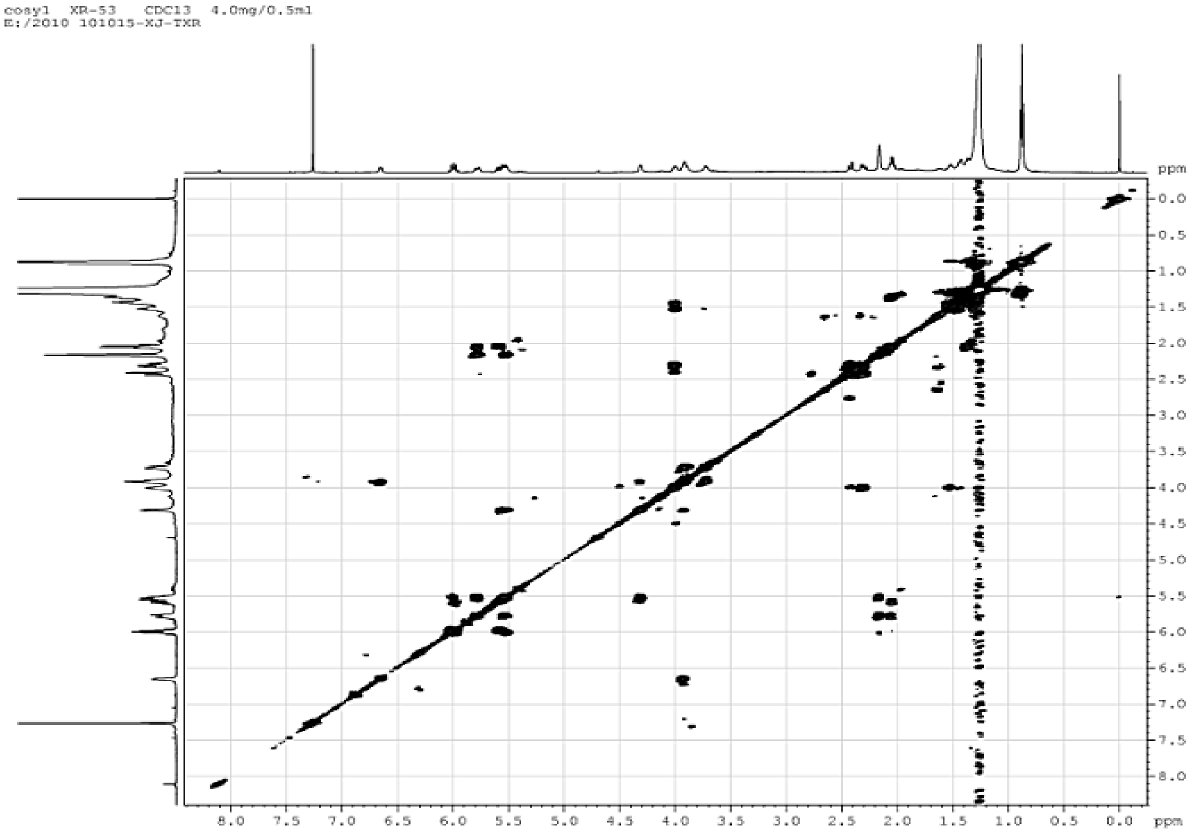

Figure S10. NOESY spectrum of compound **1**.

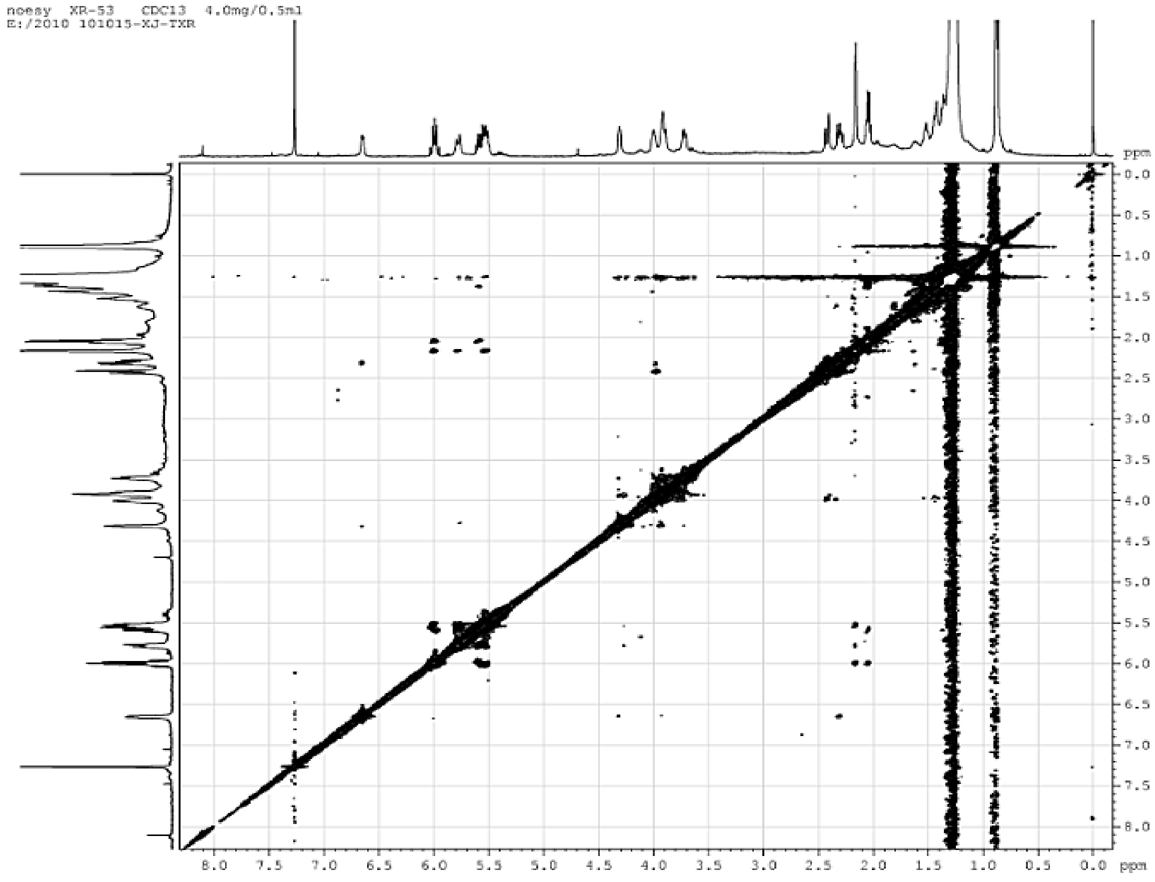

Figure S11. EI-MS data of FAME **1**.

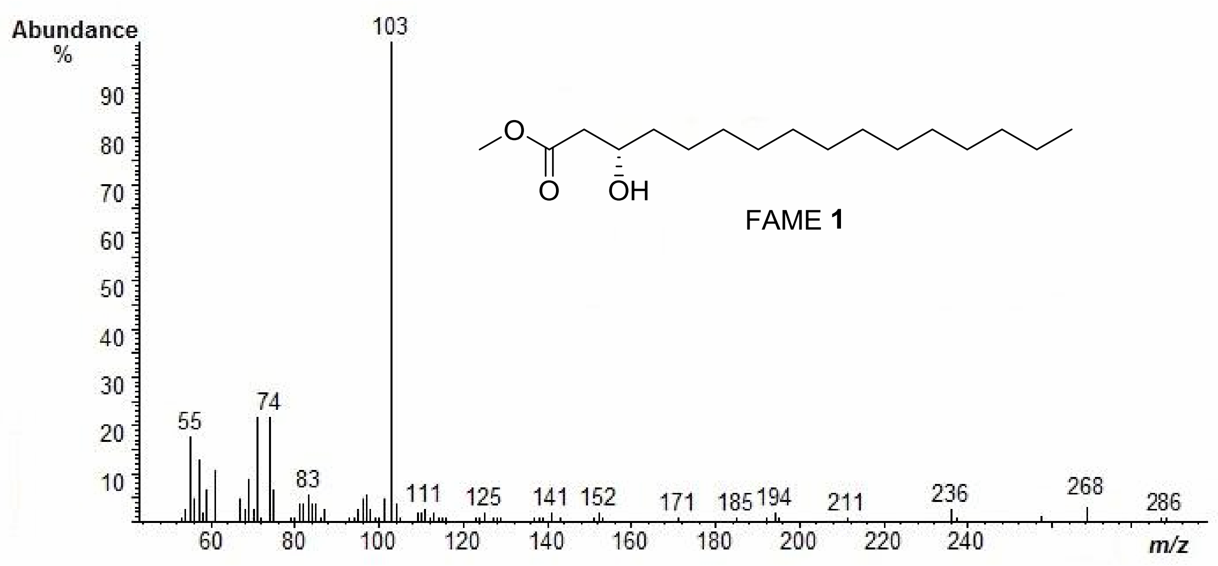

Figure S12. HR-ESI-MS (positive) spectrum of compound **2**.

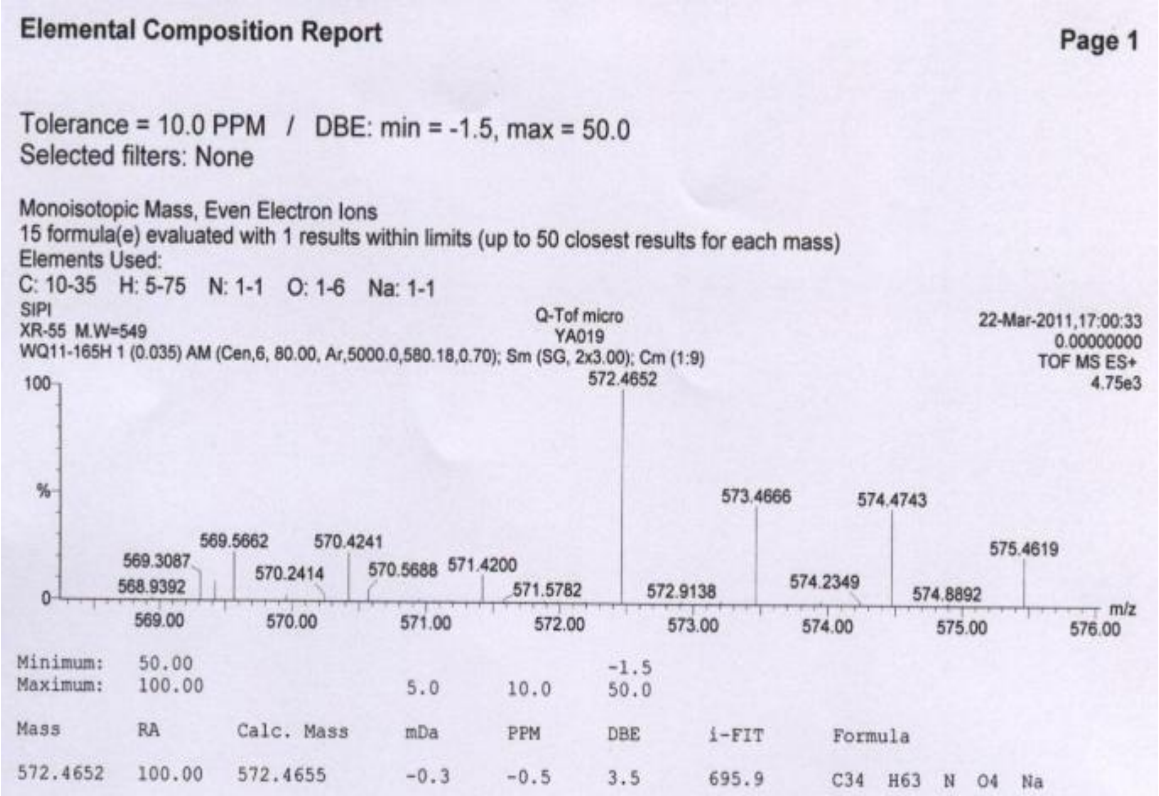

Figure S13. ESI-MS (positive and negative) spectrum of compound **2**.

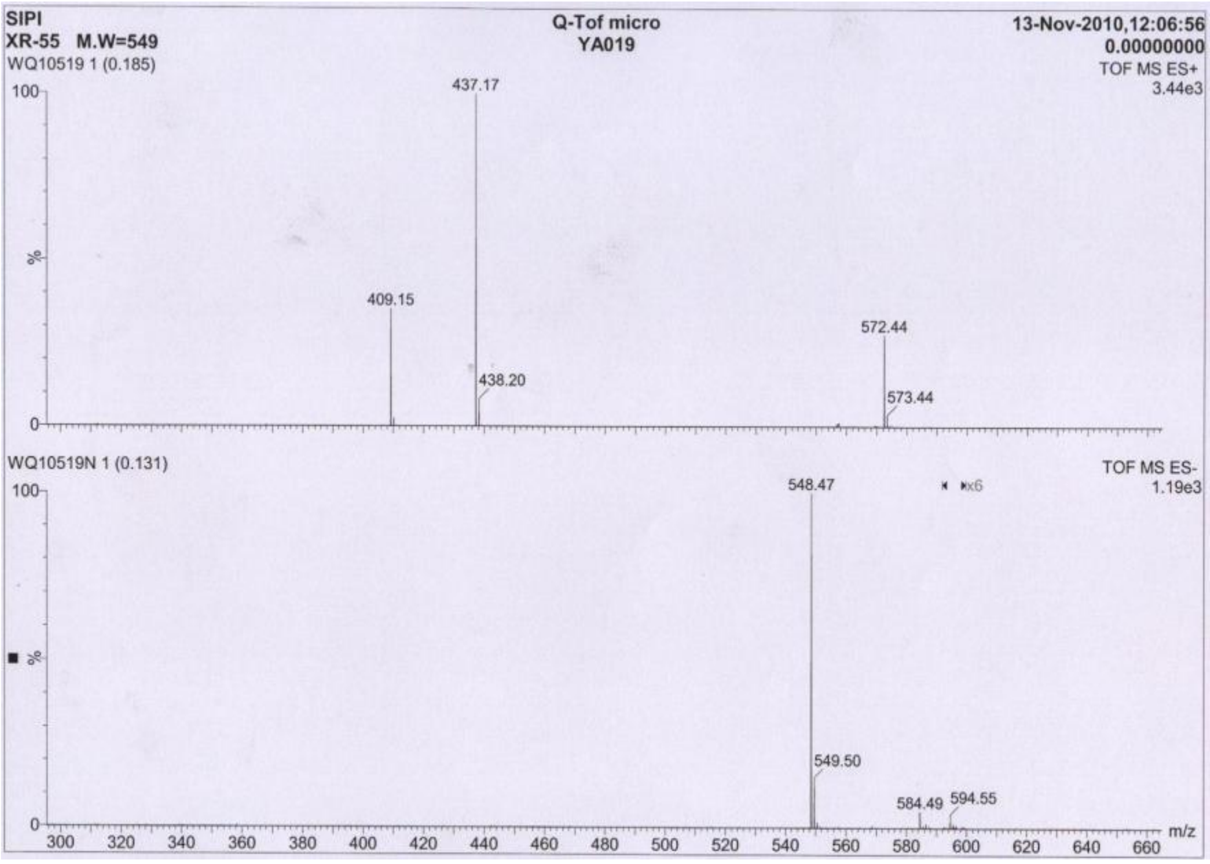

Figure S14. ESI-MS (positive and negative) fragments spectrum of compound **2.**

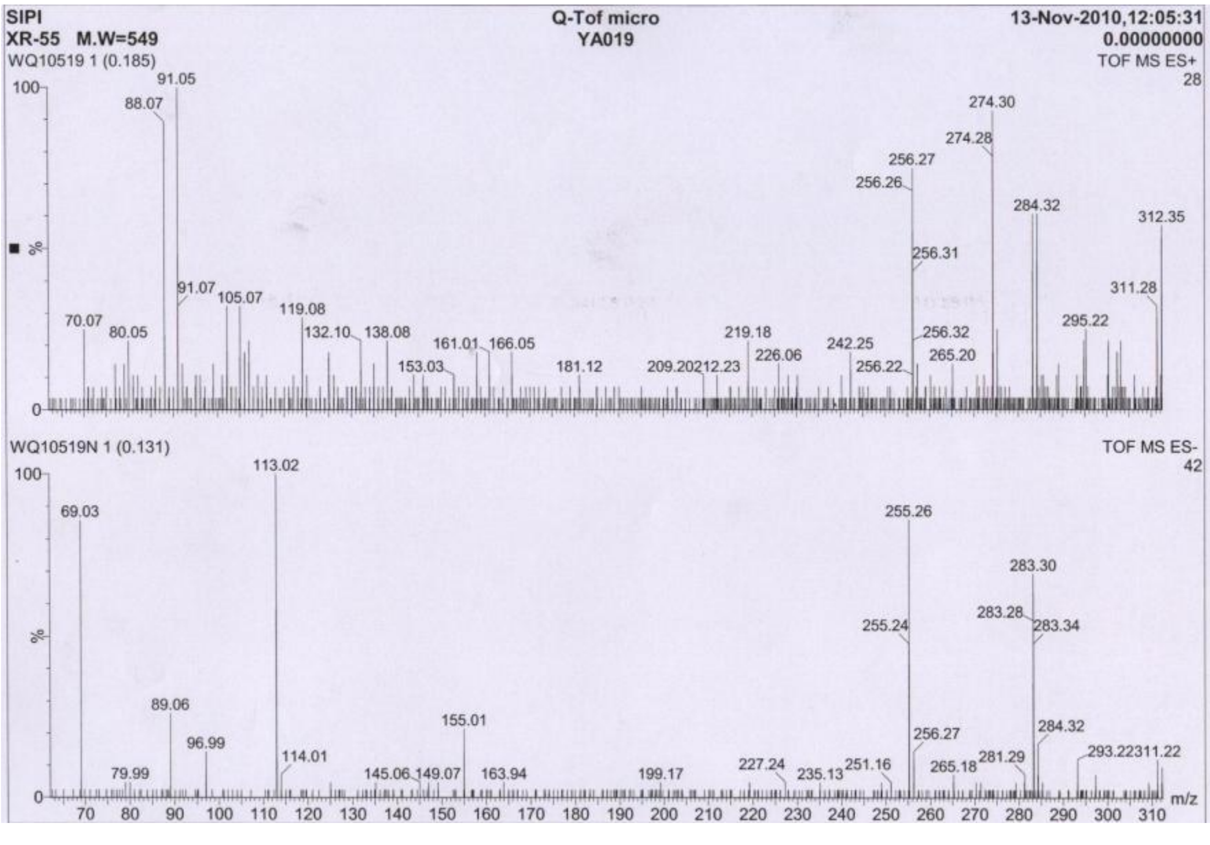

Figure S15. ^{1}H-NMR spectrum of compound **2**.

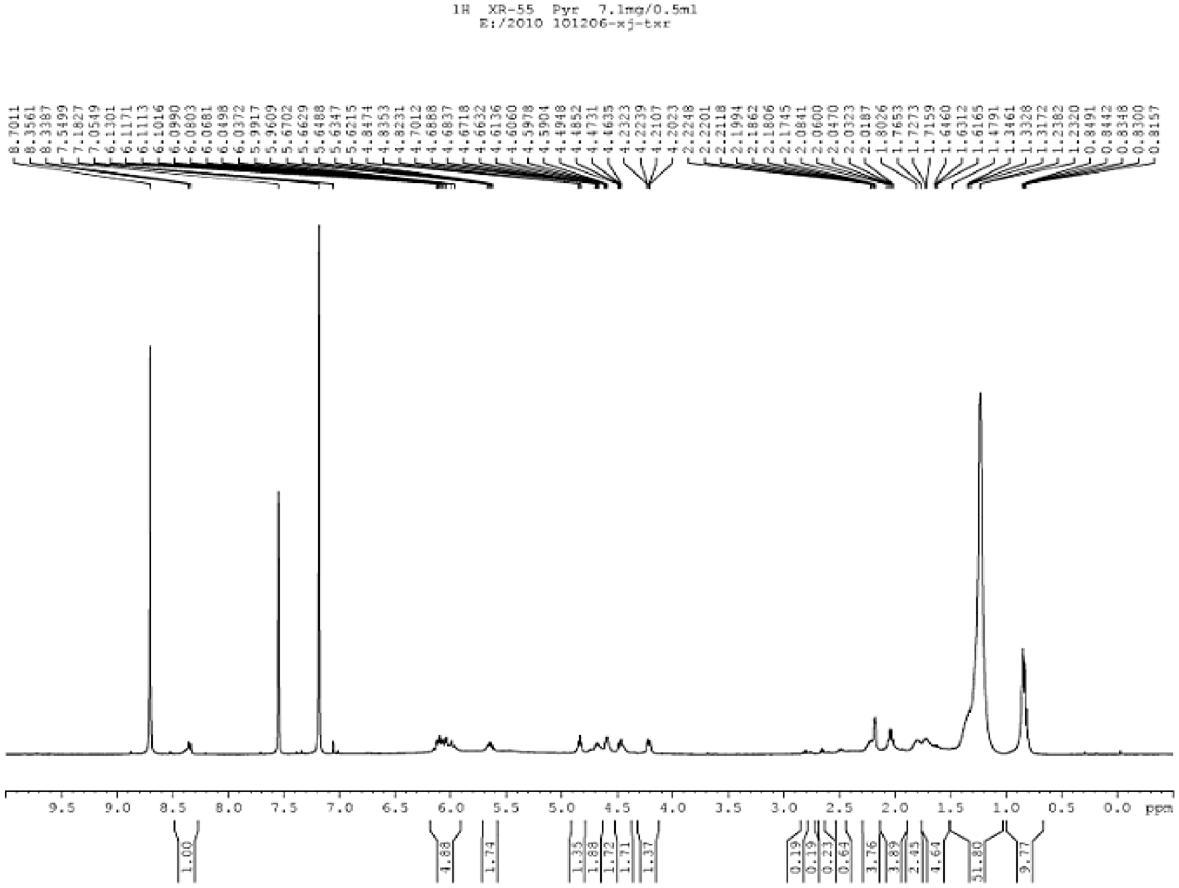

Figure S16. ^{13}C-NMR spectrum of compound **2**.

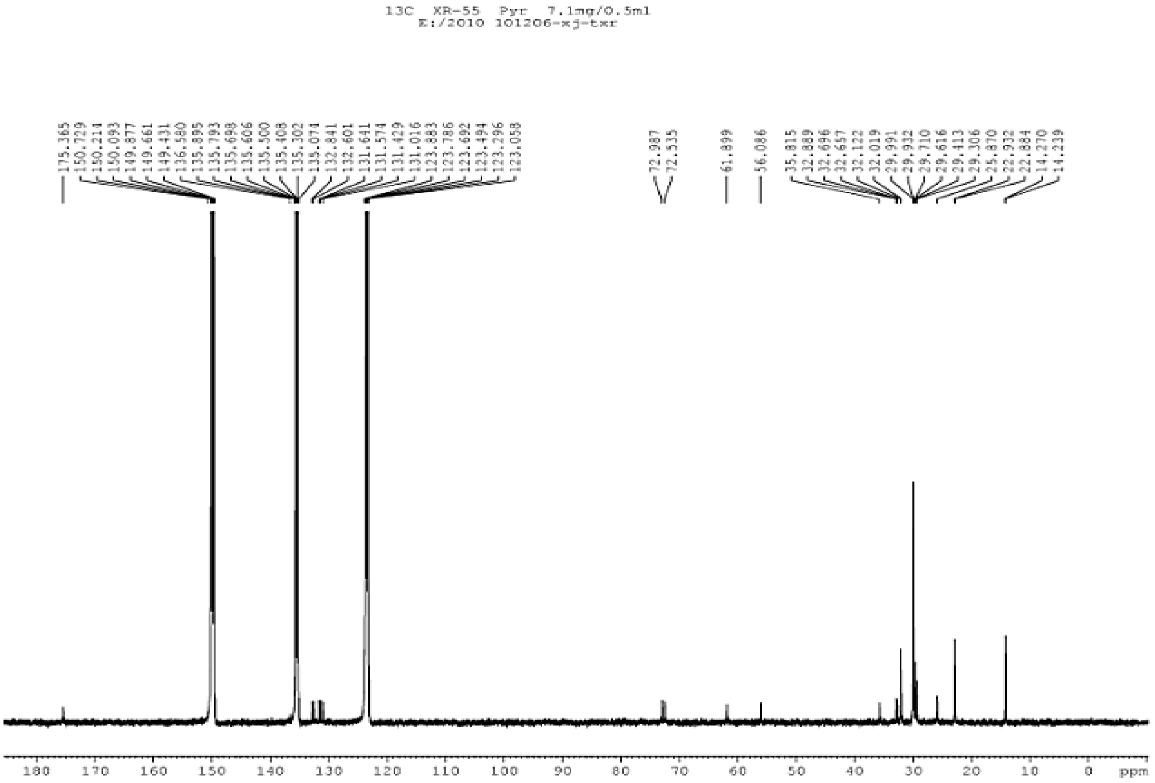

Figure S17. DEPT spectrum of compound **2**.

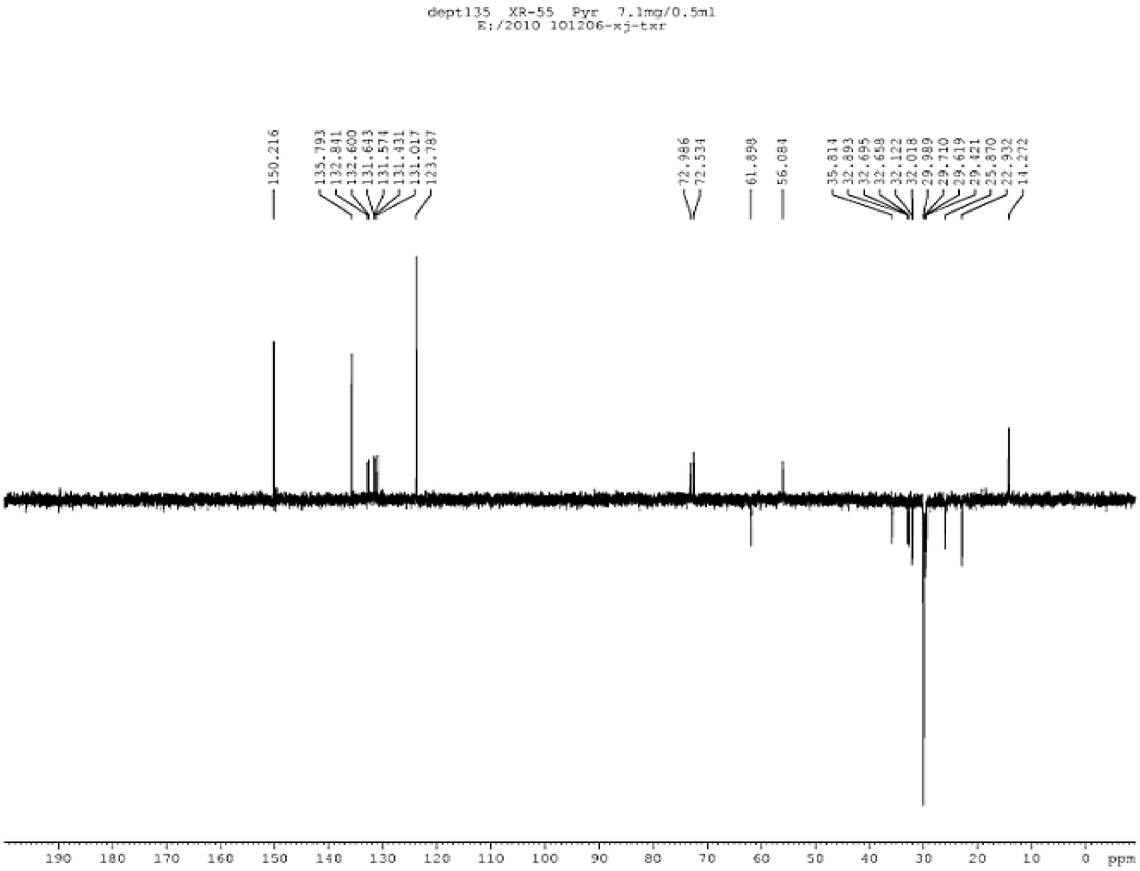

Figure S18. HSQC spectrum of compound **2**.

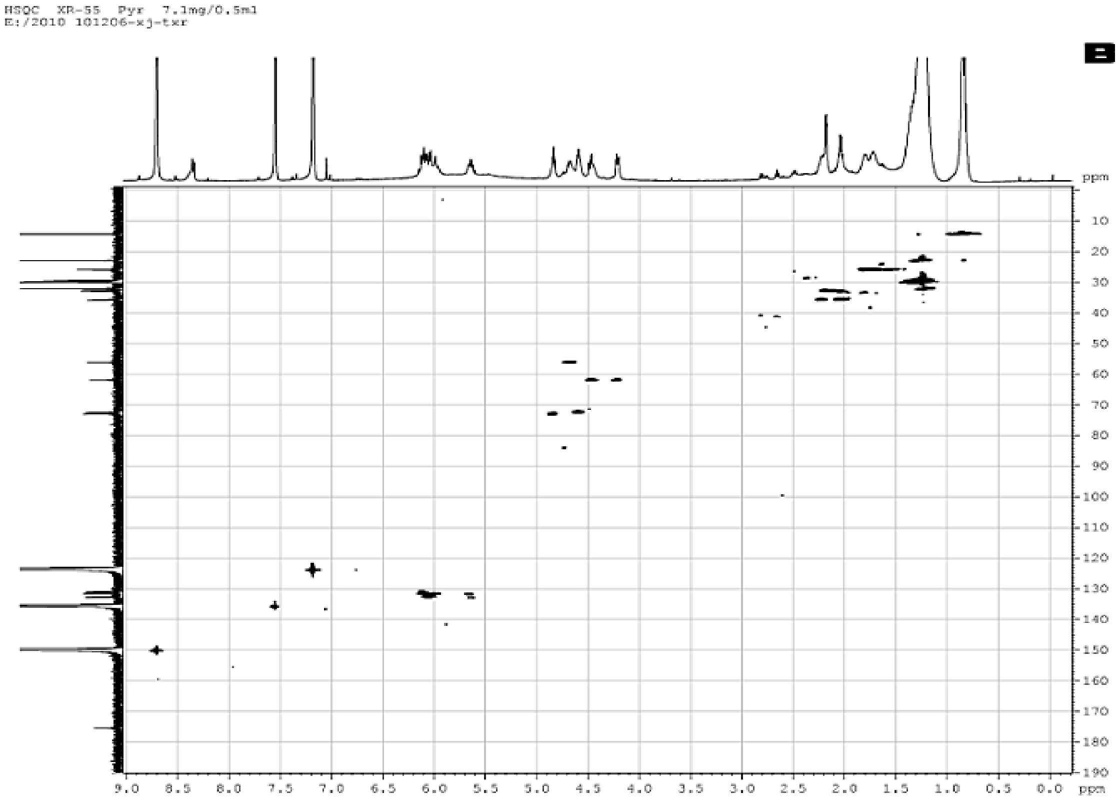

Figure S19. HMBC spectrum of compound **2**.

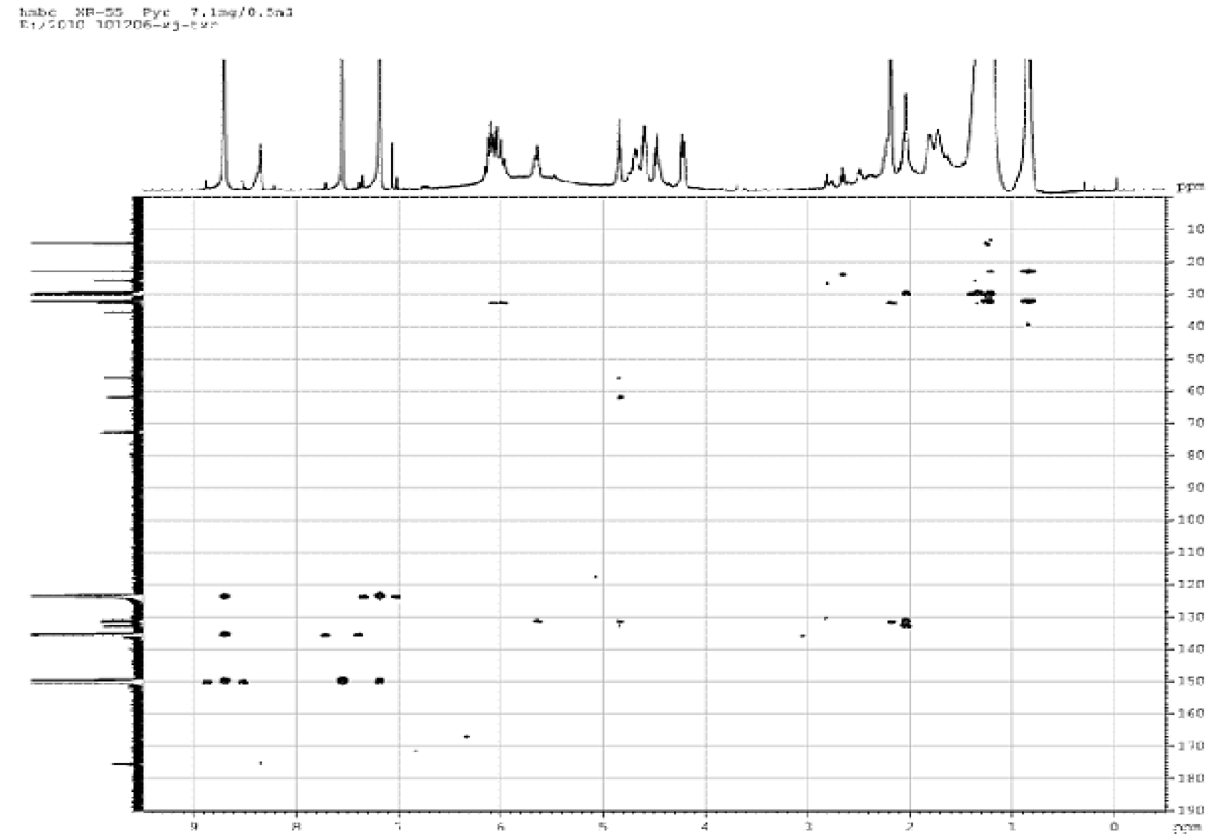

Figure S20. ^{1}H-^{1}H COSY spectrum of compound **2**.

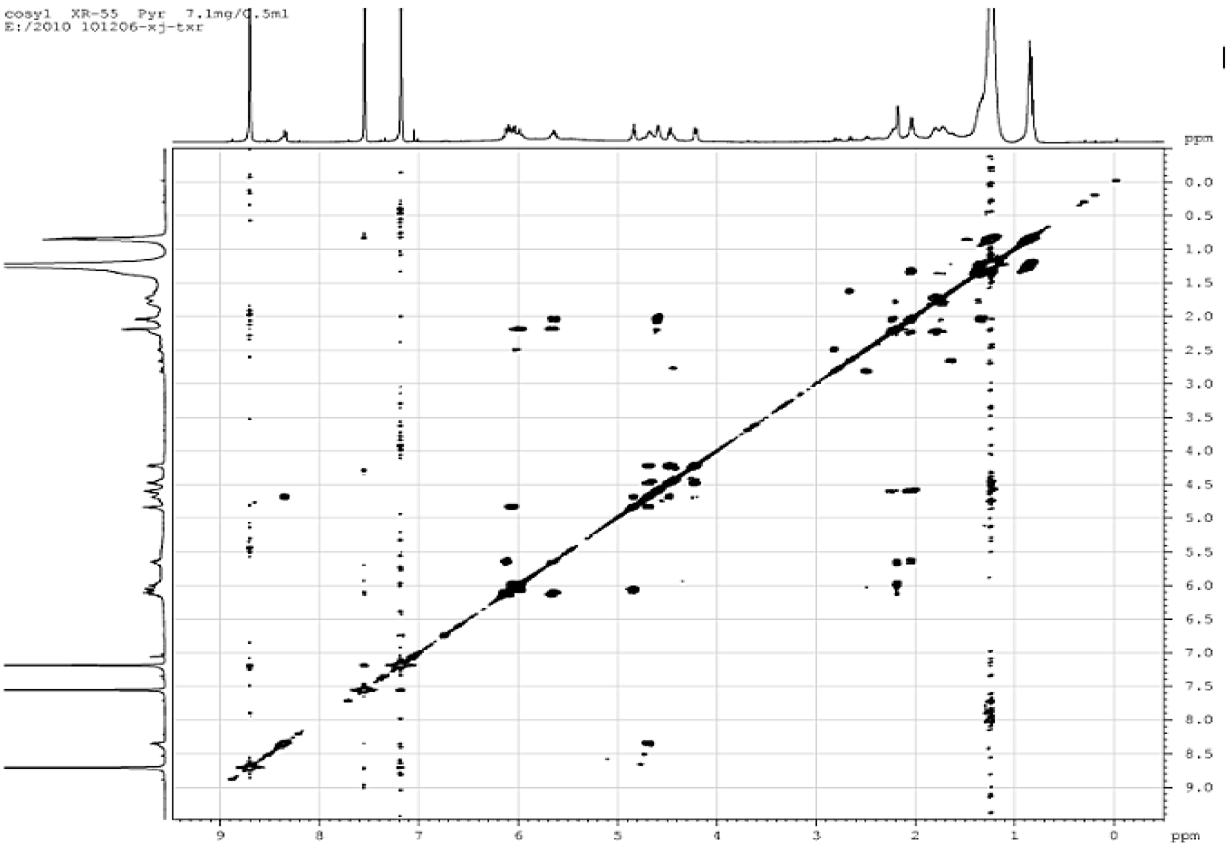

Figure S21. NOESY spectrum of compound **2**.

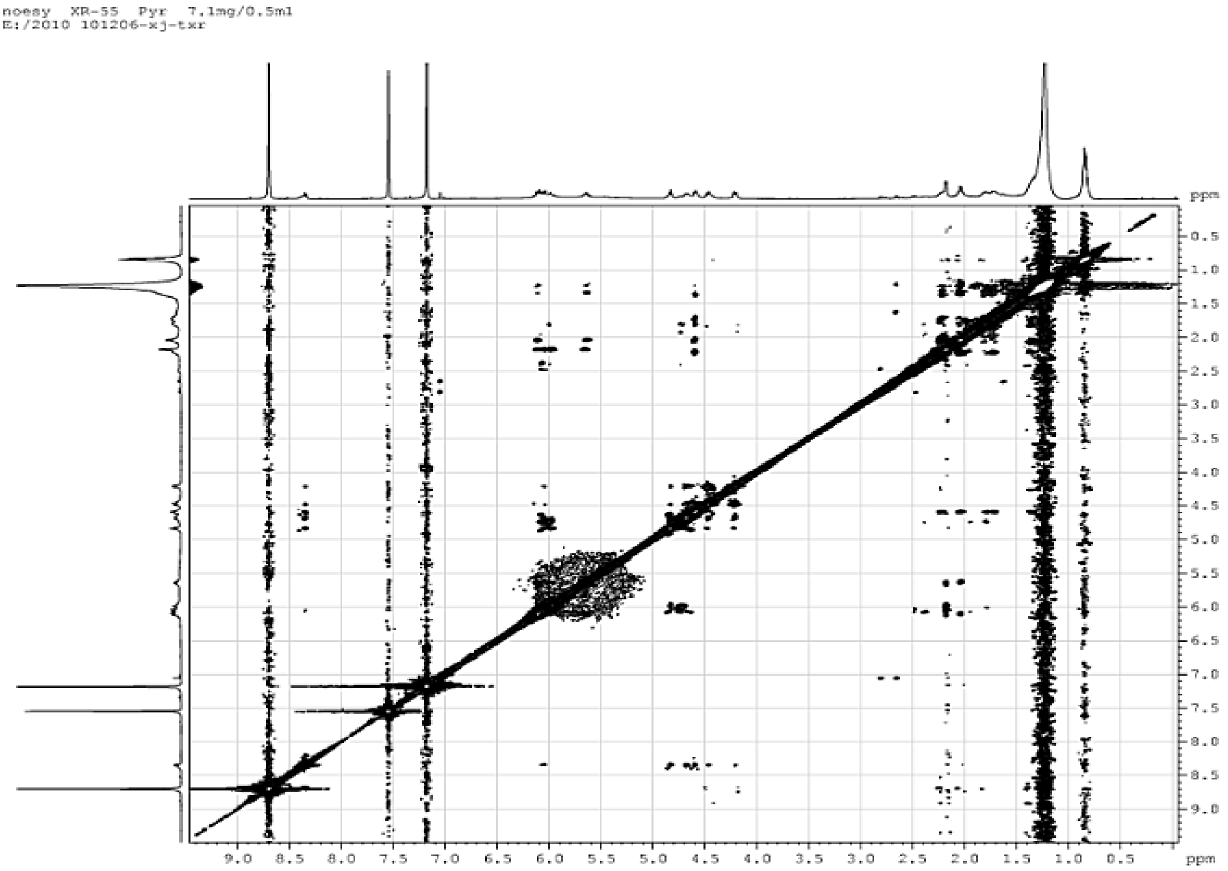

Figure S22. EI-MS data of FAME **2**.

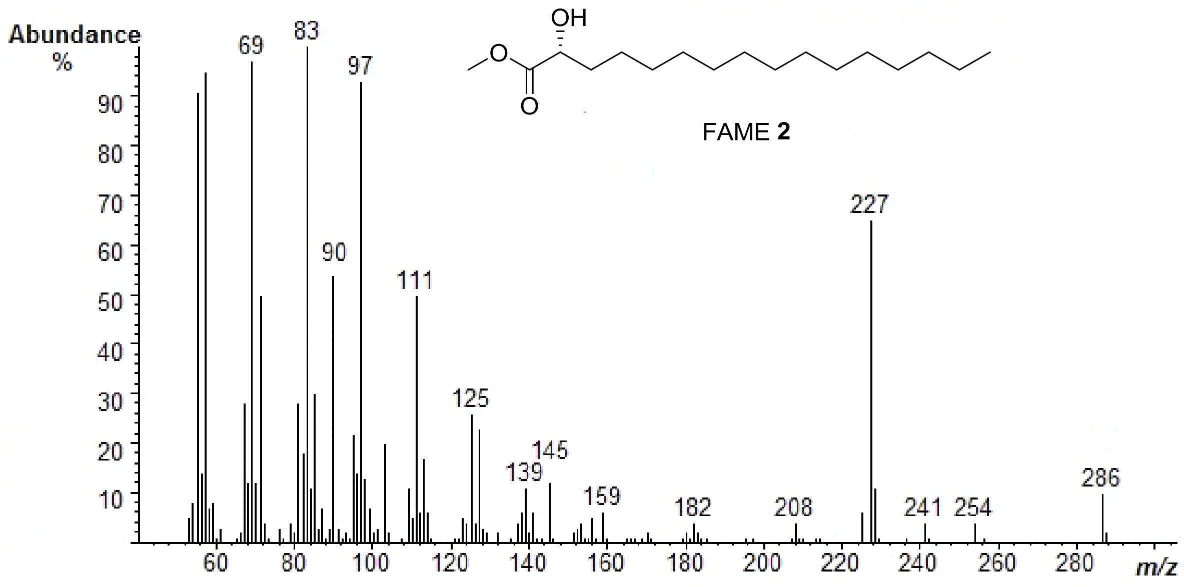

Figure S23. ESI-MS (positive and negative) spectrum of compound **3**.

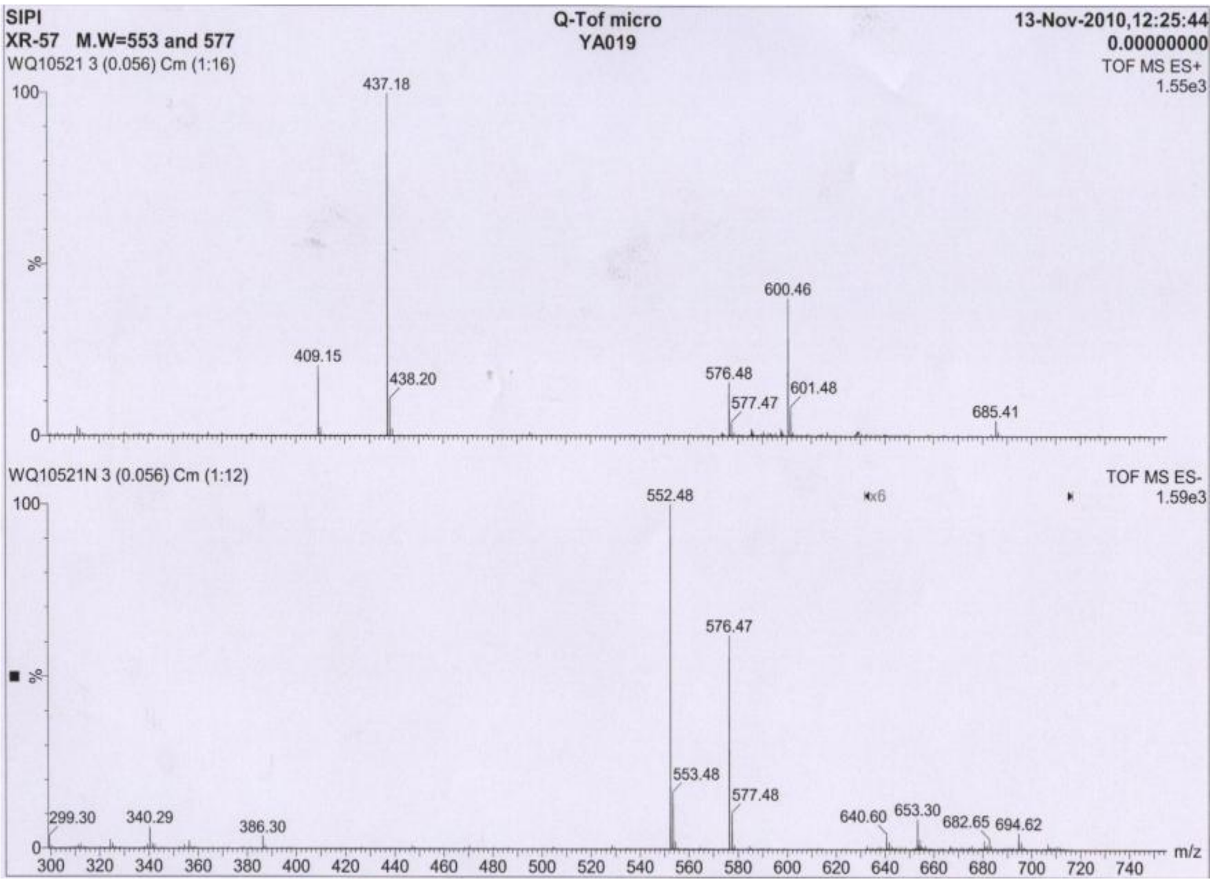

Figure S24. ESI-MS (positive and negative) fragments spectrum of compound **3**.

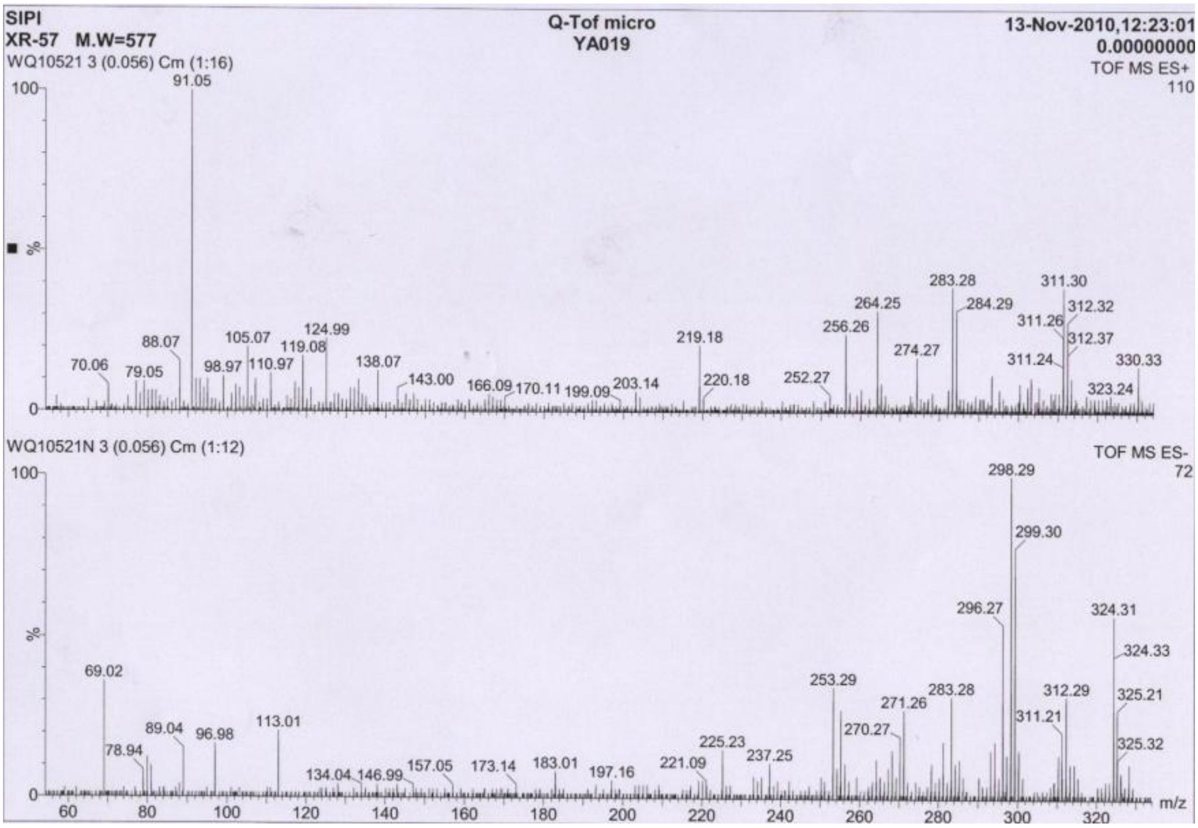

Figure S25. ^{1}H-NMR spectrum of compound **3**.

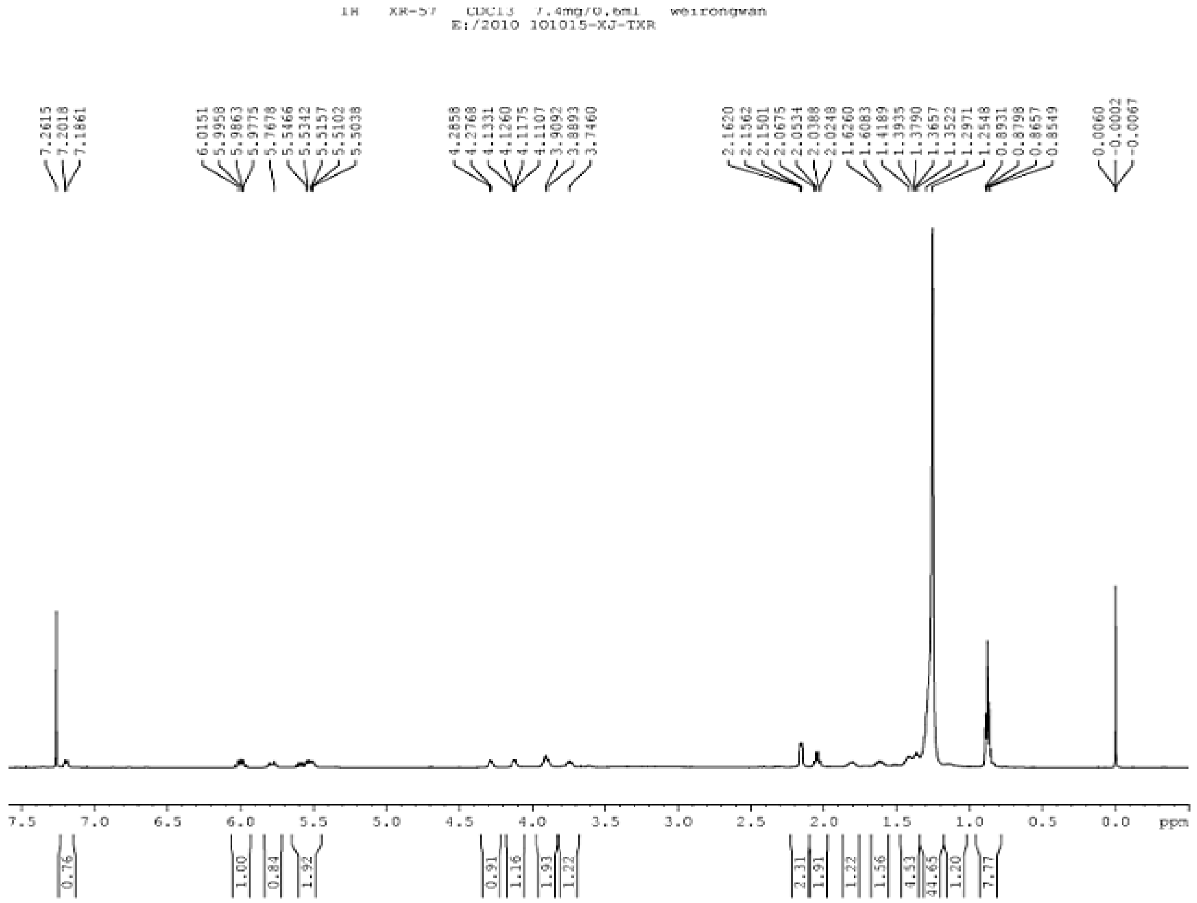

Figure S26. ^{13}C-NMR spectrum of compound **3**.

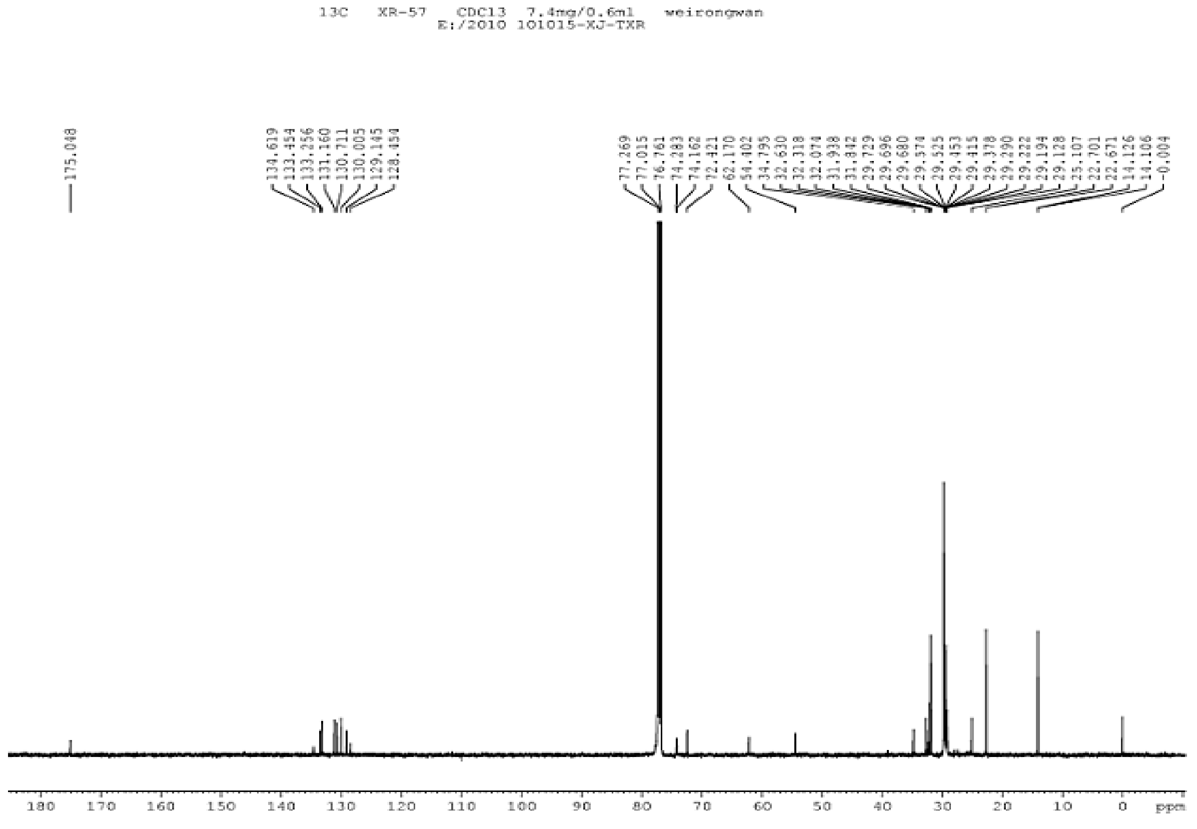

Figure S27. DEPT spectrum of compound **3**.

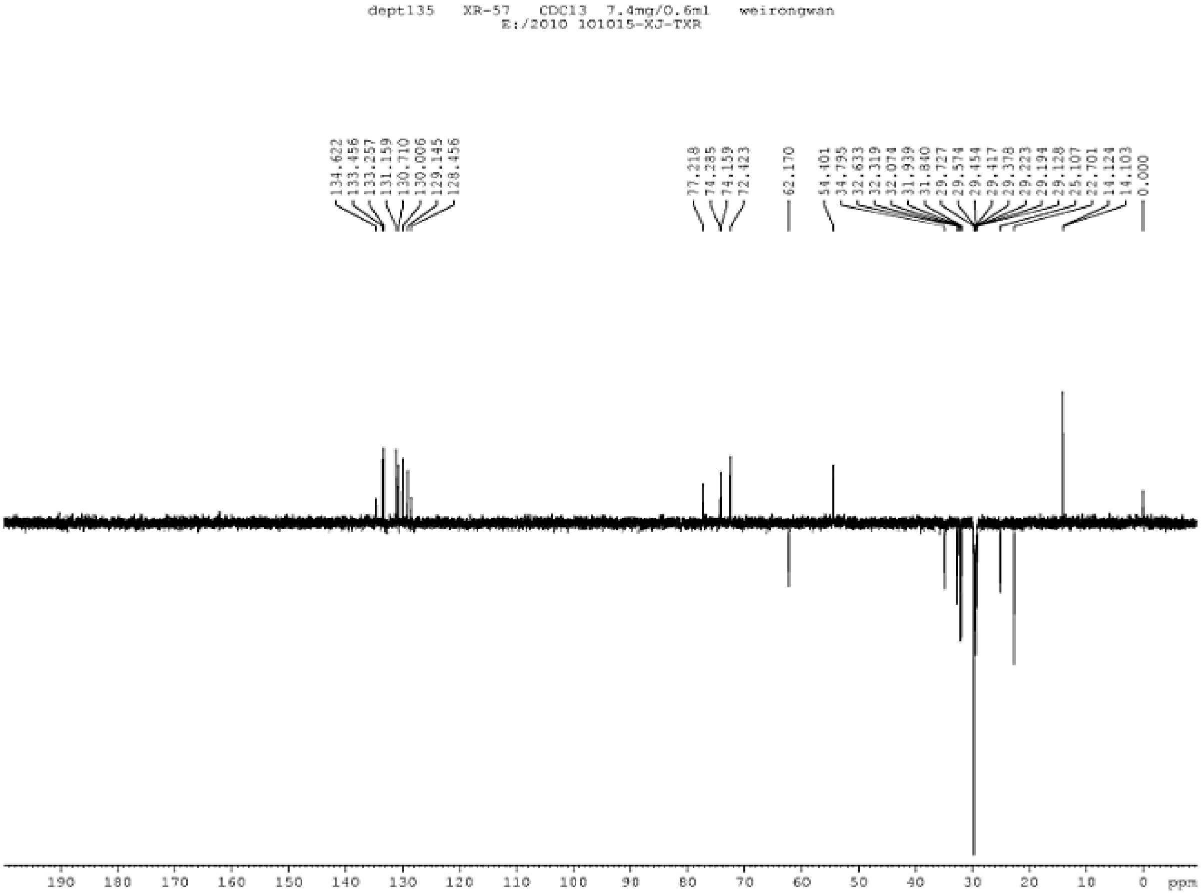

Figure S28. EI-MS data of FAME **3**.

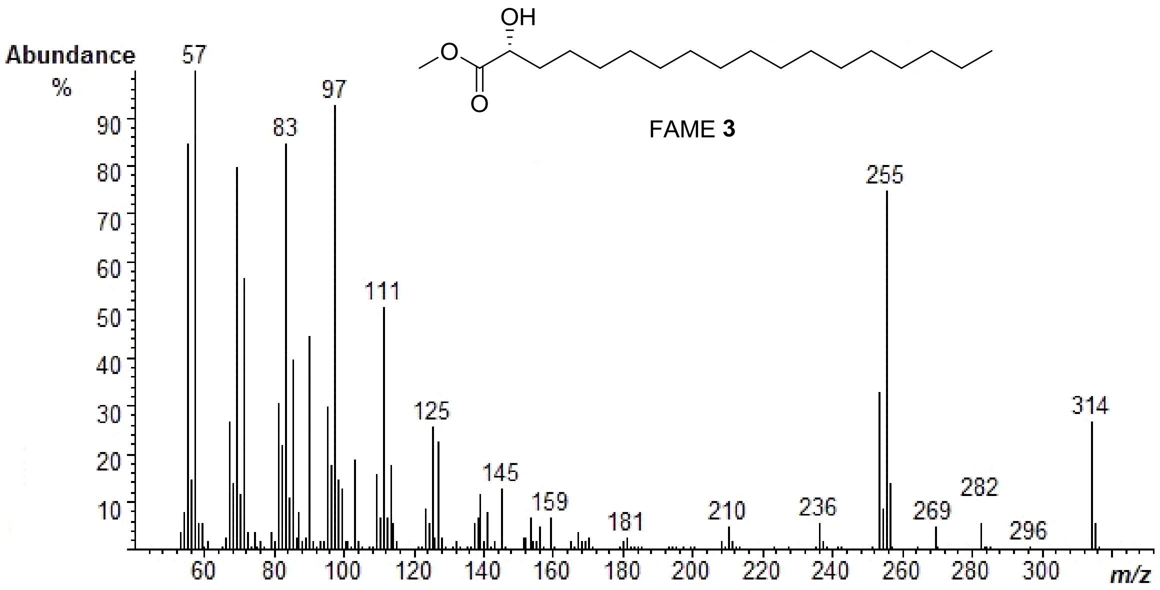

Figure S29. HR-ESI-MS (positive) spectrum of compound **4**.

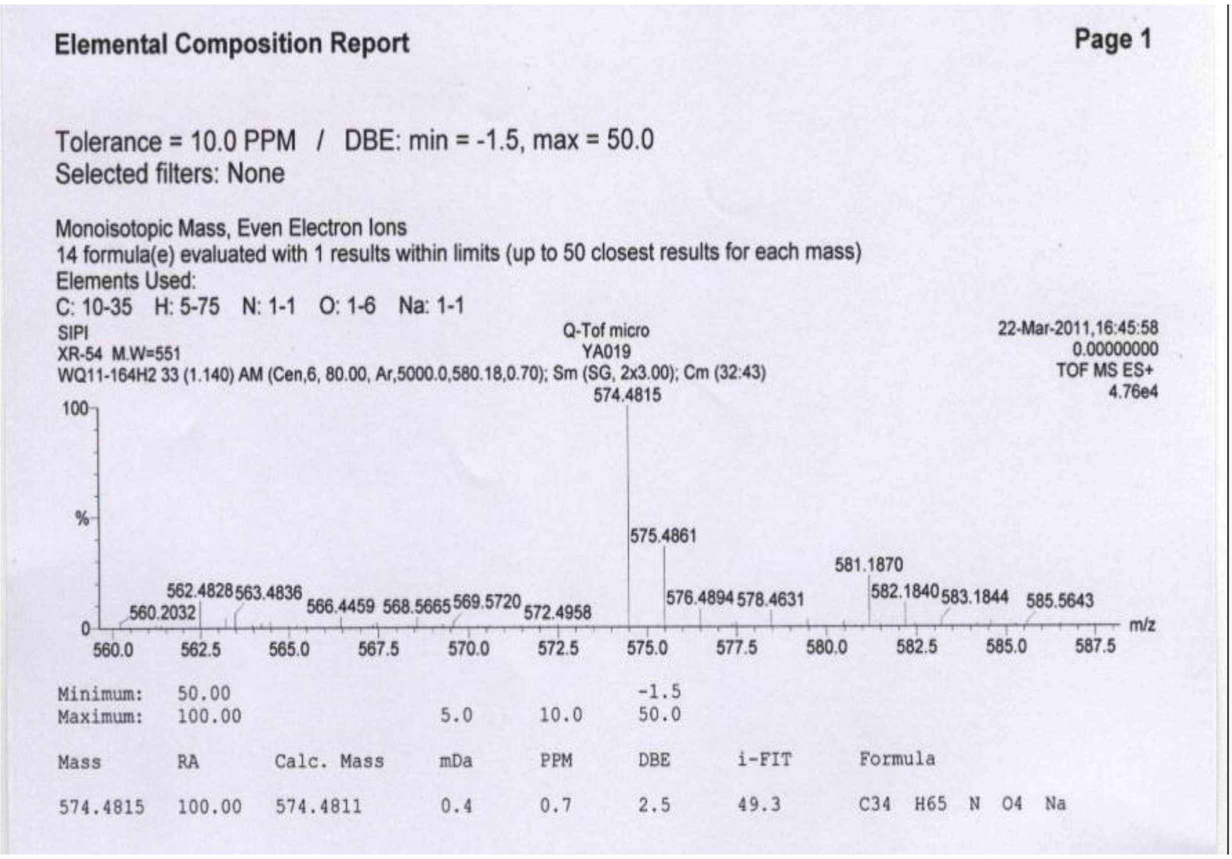

Elemental Composition Report Page 1

Tolerance = 10.0 PPM / DBE: min = -1.5, max = 50.0
Selected filters: None

Monoisotopic Mass, Even Electron Ions
14 formula(e) evaluated with 1 results within limits (up to 50 closest results for each mass)
Elements Used:
C: 10-35 H: 5-75 N: 1-1 O: 1-6 Na: 1-1

Minimum:	50.00				-1.5		
Maximum:	100.00		5.0	10.0	50.0		
Mass	RA	Calc. Mass	mDa	PPM	DBE	i-FIT	Formula
574.4815	100.00	574.4811	0.4	0.7	2.5	49.3	C34 H65 N O4 Na

Figure S30. ESI-MS (positive and negative) spectrum of compound **4**.

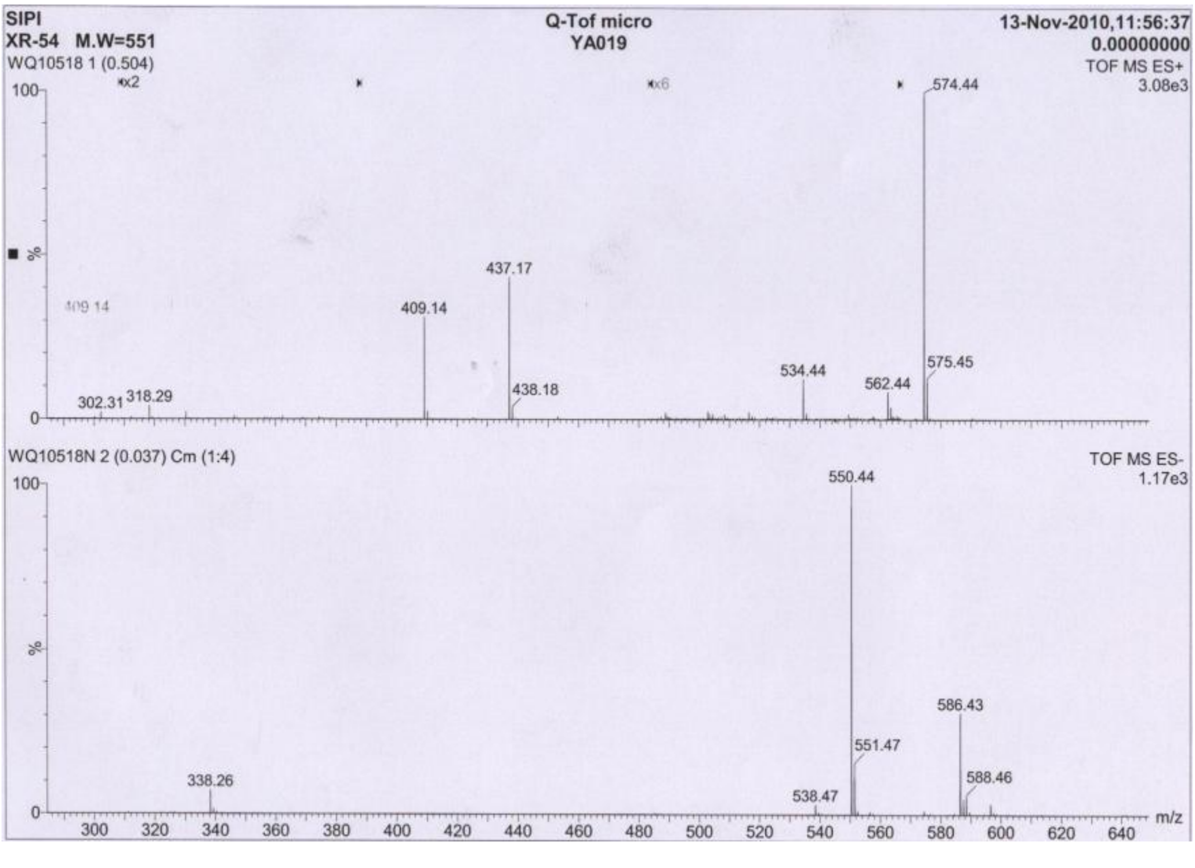

Figure S31. ESI-MS (positive and negative) fragments spectrum of compound **4**.

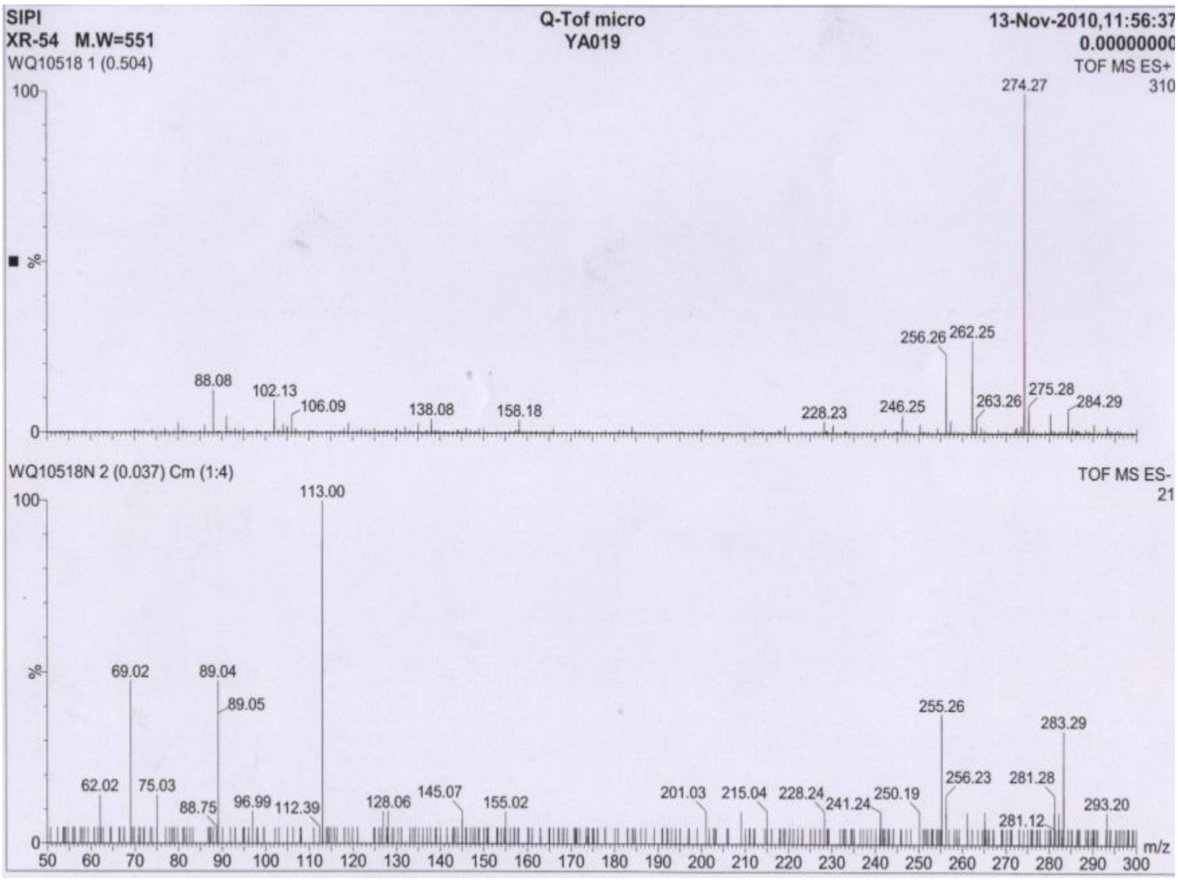

Figure S32. ^{1}H-NMR spectrum of compound **4**.

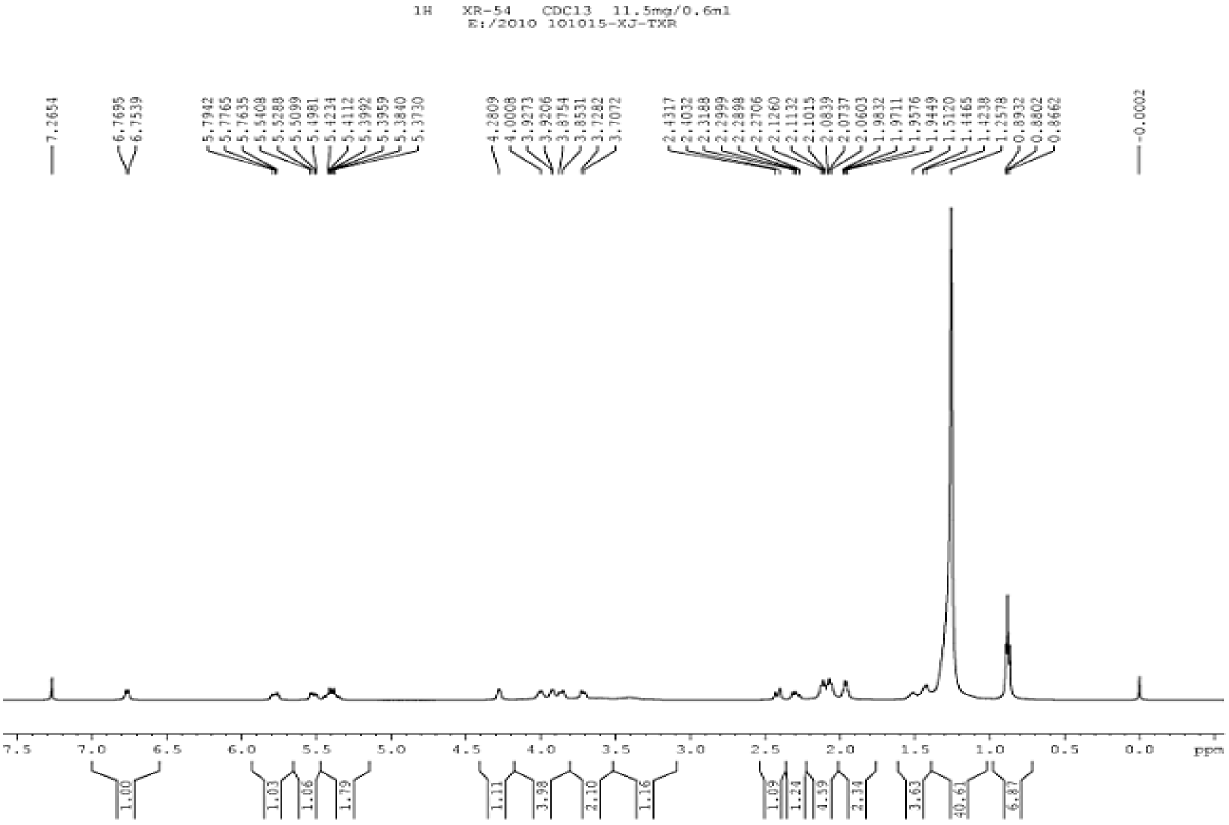

Figure S33. ^{13}C-NMR spectrum of compound **4**.

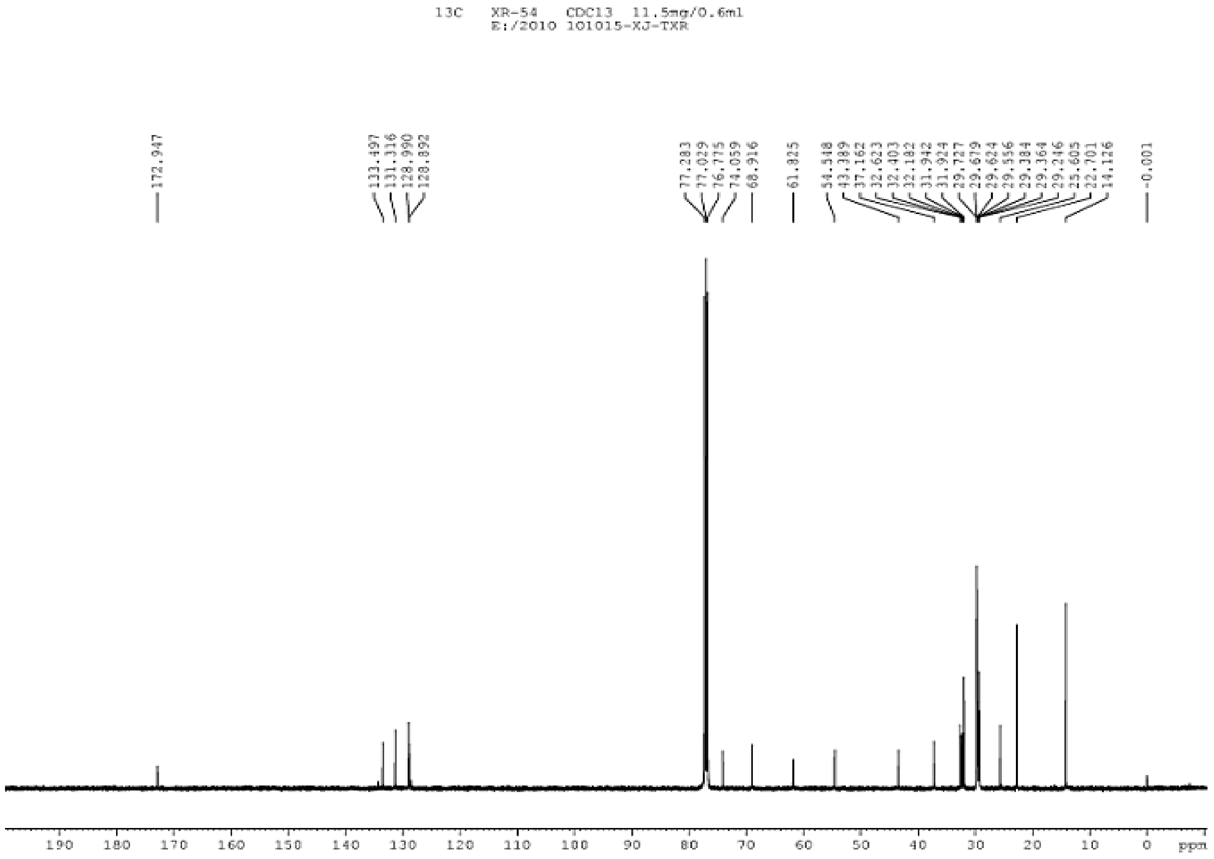

Figure S34. DEPT spectrum of compound **4**.

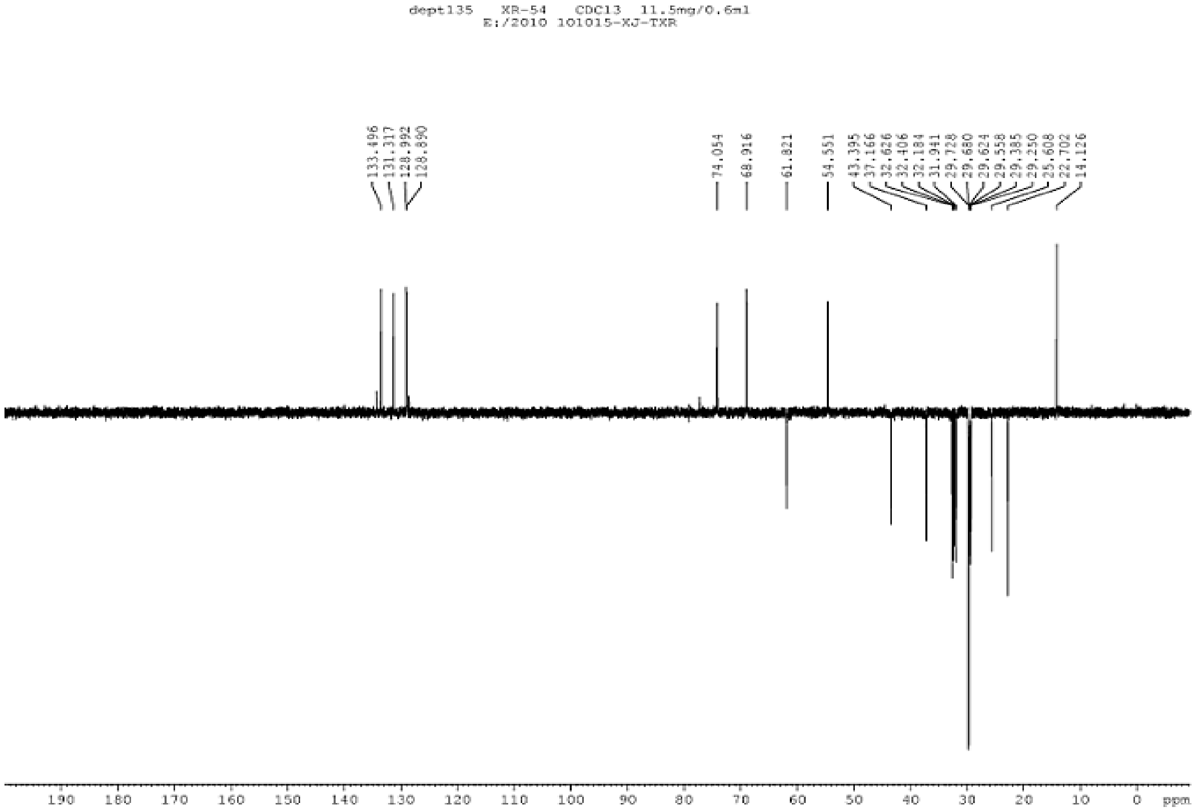

Figure S35. HR-ESI-MS (positive) spectrum of compound **5**.

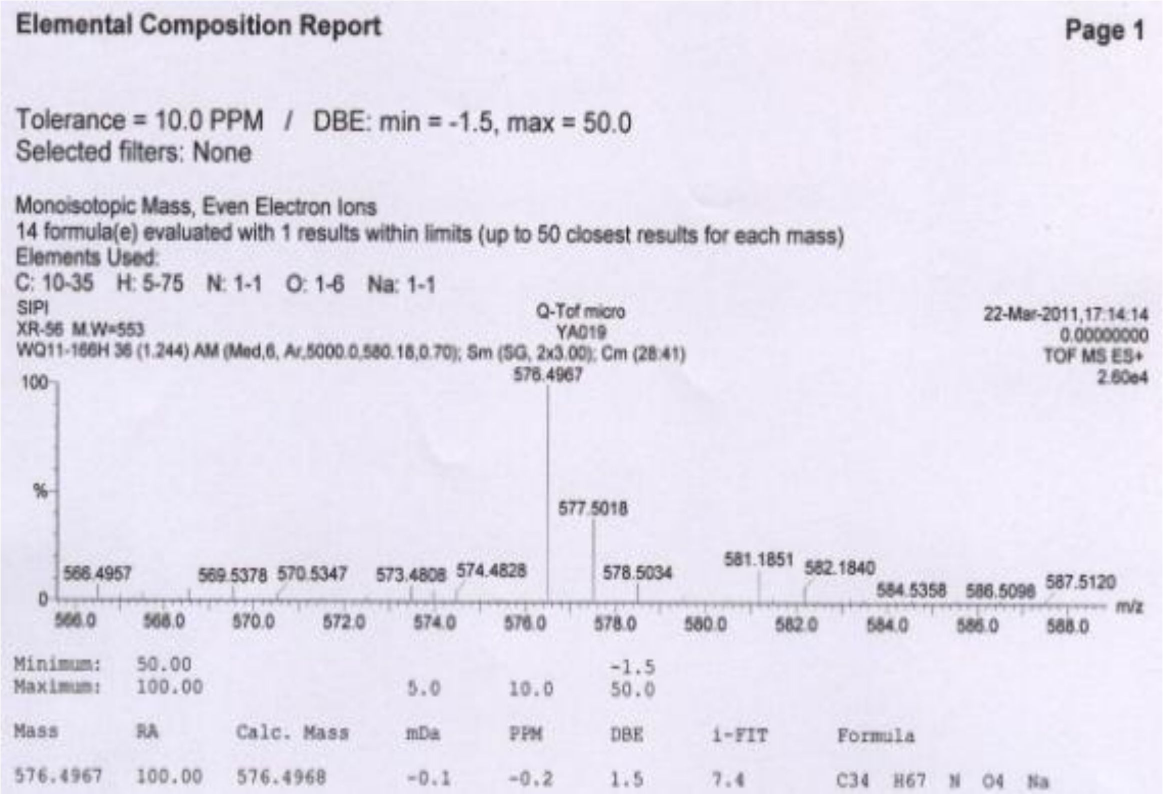

Figure S36. ESI-MS (positive and negative) spectrum of compound **5**.

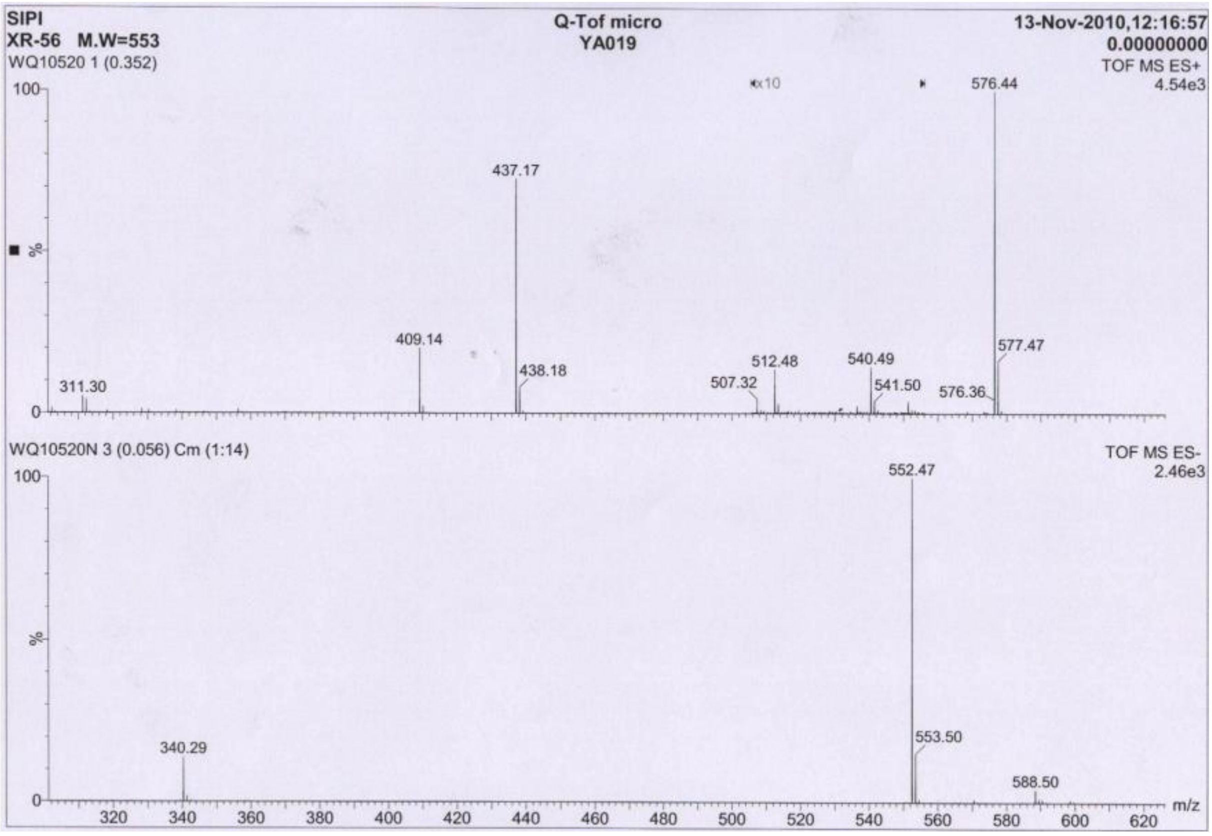

Figure S37. ESI-MS (positive and negative) fragments spectrum of compound **5**.

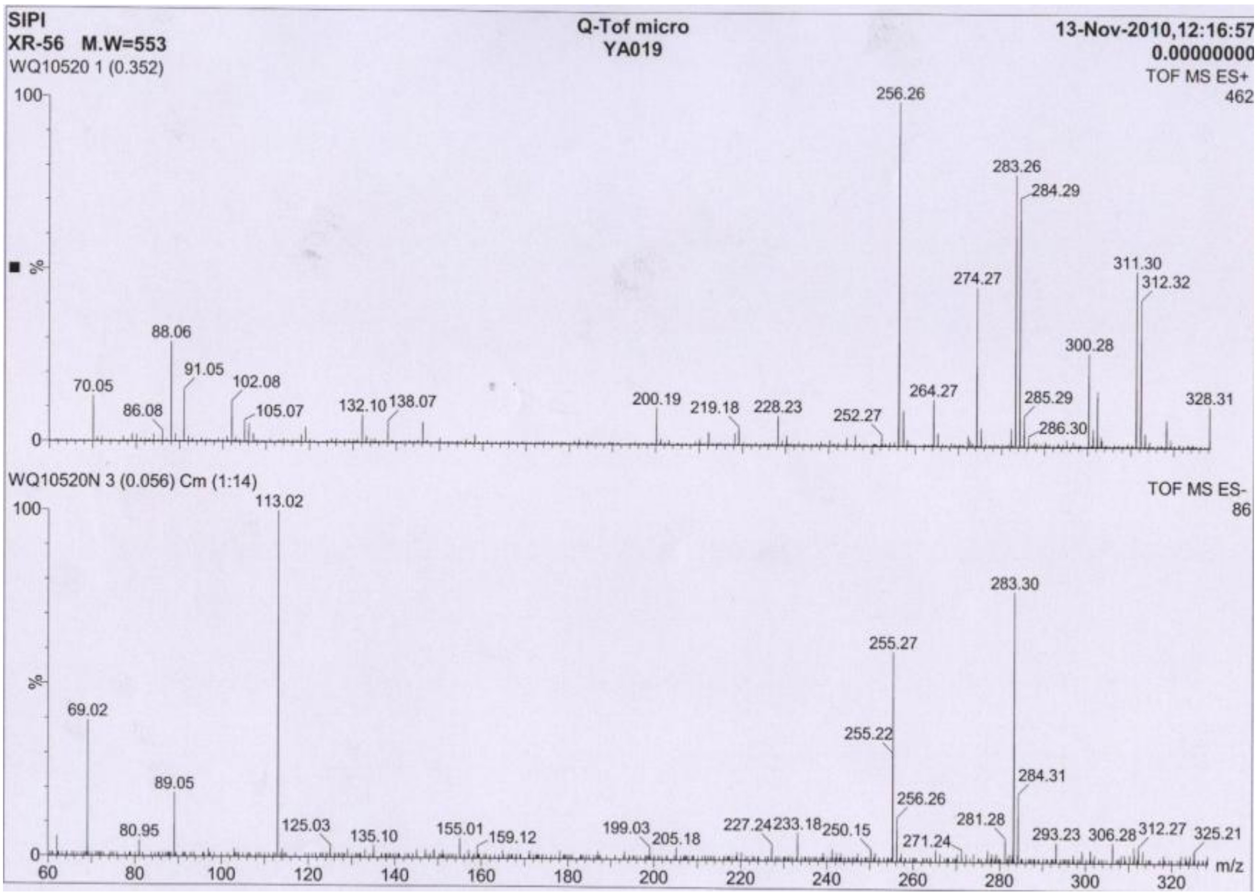

Figure S38. ^{1}H-NMR spectrum of compound **5**.

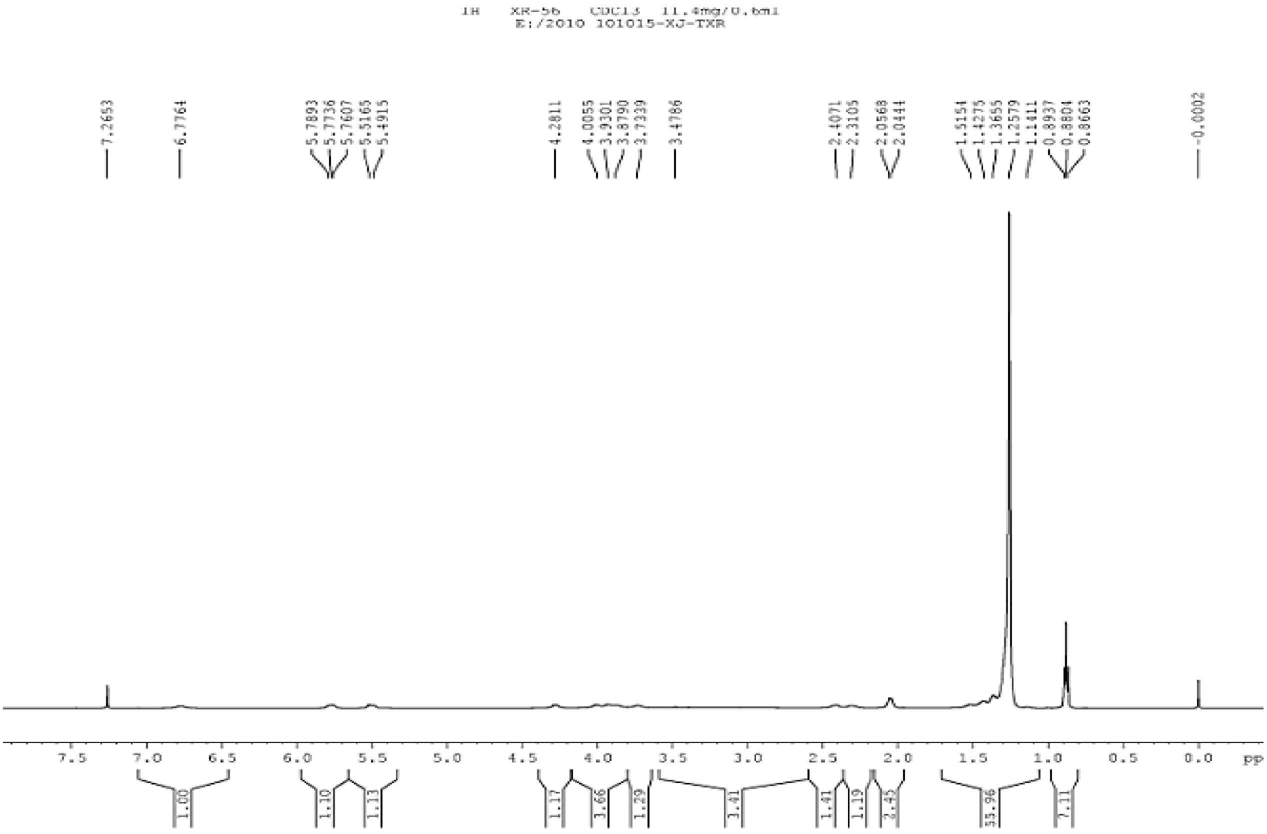

Figure S39. ^{13}C-NMR spectrum of compound **5**.

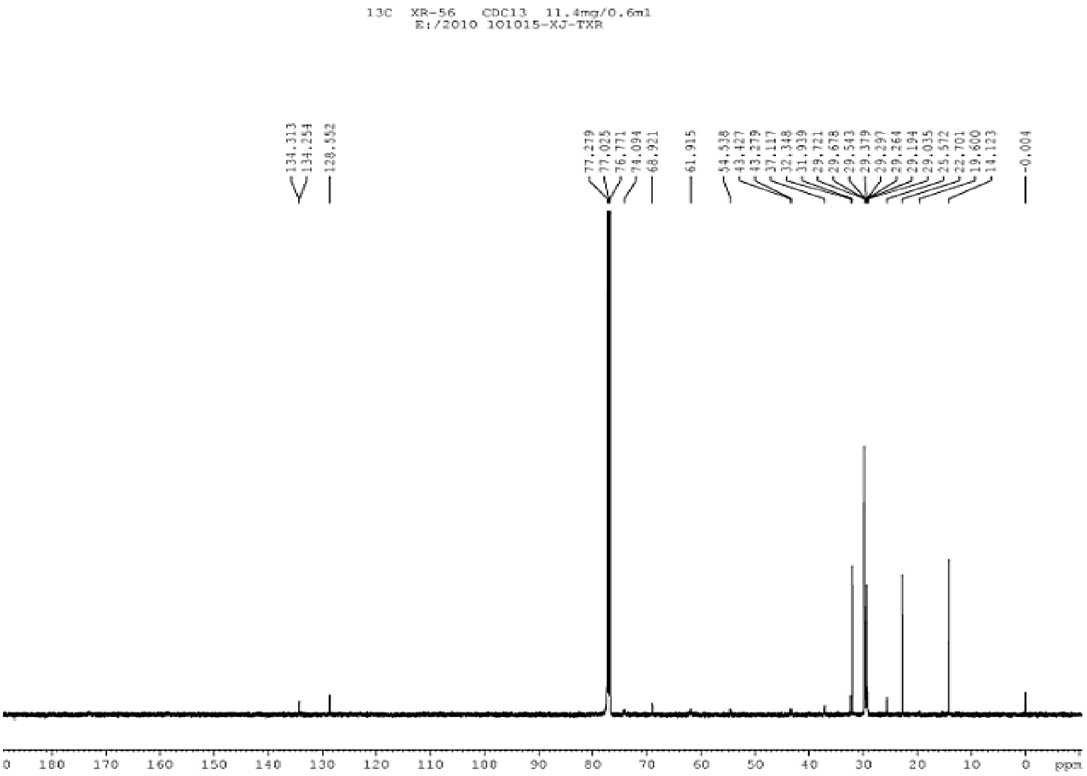

Figure S40. DEPT spectrum of compound **5**.

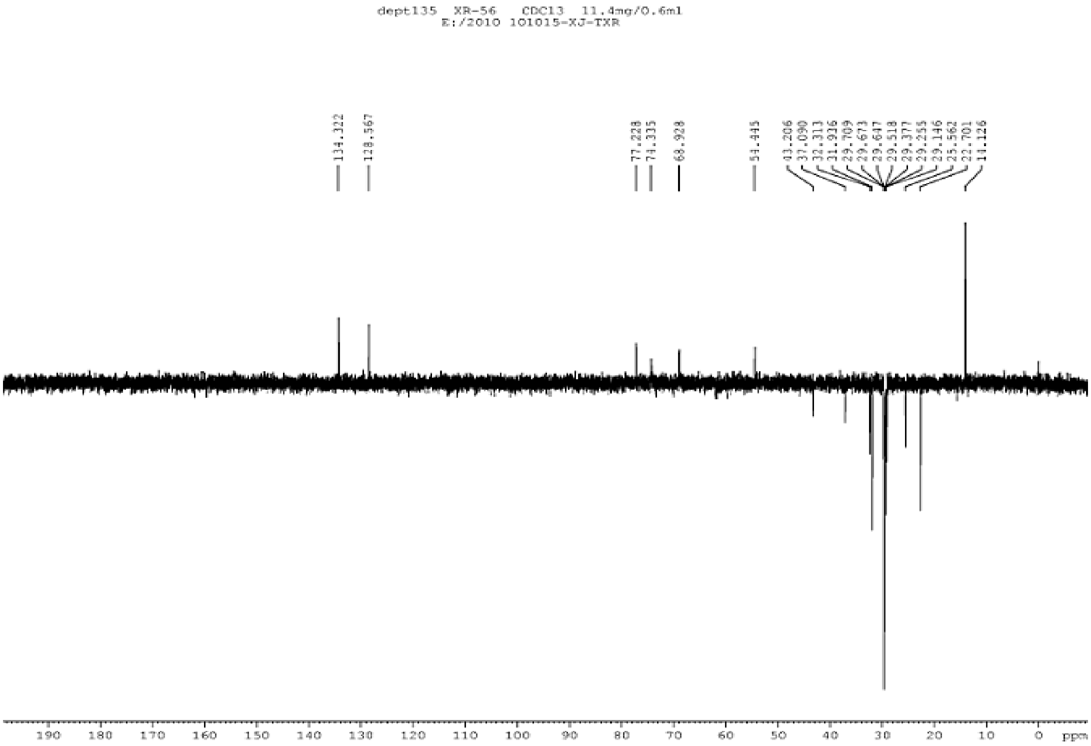

© 2014 by the authors; licensee MDPI, Basel, Switzerland. This article is an open access article distributed under the terms and conditions of the Creative Commons Attribution license (http://creativecommons.org/licenses/by/3.0/).
